# 1‐(Azidomethyl)‐5*H*‐Tetrazole: A Powerful New Ligand for Highly Energetic Coordination Compounds

**DOI:** 10.1002/chem.202200492

**Published:** 2022-05-25

**Authors:** Moritz Kofen, Marcus Lommel, Maximilian H. H. Wurzenberger, Thomas M. Klapötke, Jörg Stierstorfer

**Affiliations:** ^1^ Department of Chemistry Ludwig-Maximilian University of Munich Butenandtstrasse 5–13 81377 Munich Germany

**Keywords:** alkylazides, energetic coordination compounds, initiation, primary explosives, tetrazole

## Abstract

Highly energetic 1‐(azidomethyl)‐5*H*‐tetrazole (AzMT, **3**) has been synthesized and characterized. This completes the series of 1‐(azidoalkyl)‐5*H*‐tetrazoles represented by 1‐(azidoethyl)‐5*H*‐tetrazole (AET) and 1‐(azidopropyl)‐5*H*‐tetrazole (APT). AzMT was thoroughly analyzed by single‐crystal X‐ray diffraction experiments, elemental analysis, IR spectroscopy and multinuclear (^1^H, ^13^C, ^14^N, ^15^N) NMR measurements. Several energetic coordination compounds (ECCs) of 3d metals (Mn, Fe, Cu, Zn) and silver in combination with anions such as (per)chlorate, mono‐ and dihydroxy‐trinitrophenolate were prepared, giving insight into the coordination behavior of AzMT as a ligand. The synthesized ECCs were also analyzed by X‐ray diffraction experiments, elemental analysis, and IR spectroscopy. Differential thermal analysis for all compounds was conducted, and the sensitivity towards external stimuli (impact, friction, and ESD) was measured. Due to the high enthalpy of formation of AzMT (+654.5 kJ mol^−1^), some of the resulting coordination compounds are extremely sensitive, yet are able to undergo deflagration‐to‐detonation transition (DDT) and initiate pentaerythritol tetranitrate (PETN). Therefore, they are to be ranked as primary explosives.

## Graphical abstract

**Shortening the alkyl chain** in 1‐(Azidoethyl)‐5*H*‐tetrazole (AET) to a methylene bridge results in the formation of highly sensitive 1‐(azidomethyl)‐5*H*‐tetrazole (AzMT), which exhibits a greatly increased enthalpy of formation, hence a drastically increased detonation performance is calculated. Like AET, AzMT can be applied as ligand in the energetic coordination compounds (ECC) of several transition metals, showing the characteristics of primary explosives capable of initiating pentaerythritol tetranitrate. The ligand as well as all ECCs are fully characterized.

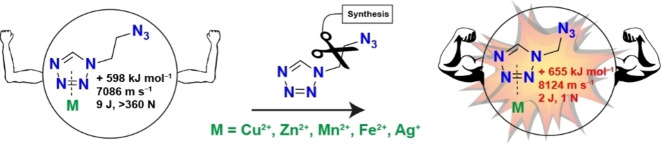

## Introduction

The design of novel energetic materials is mostly subjected to a purely synthetic approach, generally delimited by the same synthetic strategies. While some of those strategies are known and have been applied since the early 19th century, such as the introduction of several nitro groups to a molecular framework,[^1^, ^2^, ^3^, ^4^, ^5^] the concept of using energetic materials as ligands for energetic coordination compounds (ECCs) is relatively new.[^6^, ^7^] By combining a variety of metal cations (Cu^2+^, Fe^2+^, Zn^2+^, Mn^2+^, Ag^+^) with energetic anions (e. g., NO_3_^−^, ClO_3_^−^, ClO_4_^−^, N_3_^−^, and nitrophenolates) as well as a practically unlimited number of energetic ligands, ECCs offer a great adjustability and tuning of the energetic properties. It therefore expands the traditional approaches of synthesizing energetic materials, now used as ligands, by the possibility of further tuning the properties by the formation of coordination compounds.

While the tetrazole moiety, with its high nitrogen content, has been introduced in several recent high‐performing energetic materials[^8^, ^9^, ^10^, ^11^, ^12^, ^13^, ^14^] as well as potential pharmaceuticals[^15^, ^16^, ^17^] several 1 *N*‐functionalised tetrazoles^[18]^ such as 1‐methyl‐5*H*‐tetrazole^[19]^ (1‐MTZ) and 1‐amino‐5*H*‐tetrazole^[20]^ (1‐AT) promoted the range of adjustability of energetic properties of ECCs. 1‐MTZ is able to stabilize sensitive materials (e. g., AgCNO), thus performs well when desensitizing an energetic coordination compound, whereas 1‐AT drastically increases the sensitivity. Due to the exceptional high enthalpy of formation (5059 kJ kg^−1^)^[21]^ and low oxidative resistance of the 1‐AT molecule, the corresponding ECCs are among the most sensitive compounds, some being on par with pure copper(II) azide in terms of sensitivity. Efforts to find the perfect balance between performance and sensitivity has led to the formation of ECCs with 1‐(azidopropyl)‐5*H*‐tetrazole^[22]^ and 1‐(azidoethyl)‐5*H*‐tetrazole^[23]^ as ligands. Both compounds exhibit lower gas‐phase enthalpies of formation (APT: 3990 kJ kg^−1^, AET: 4616 kJ kg^−1^) and lower sensitivities (APT: >40 J, >360 N; AET: 9 J, >360 N) towards impact and friction than pure 1‐AT (<1 J, 64 N). By comparing ECCs of copper(II) and iron(II) perchlorate of 1‐AT, AET, and APT, a clear success in lowering the sensitivities can be demonstrated. The high sensitivities of both metal perchlorate complexes with 1‐AT (<1 J, <0.1 N), can be decreased by applying AET (1–3 J, 4–15 N) or APT (3 J, 24–28 N) as ligand.

**Figure 1:**
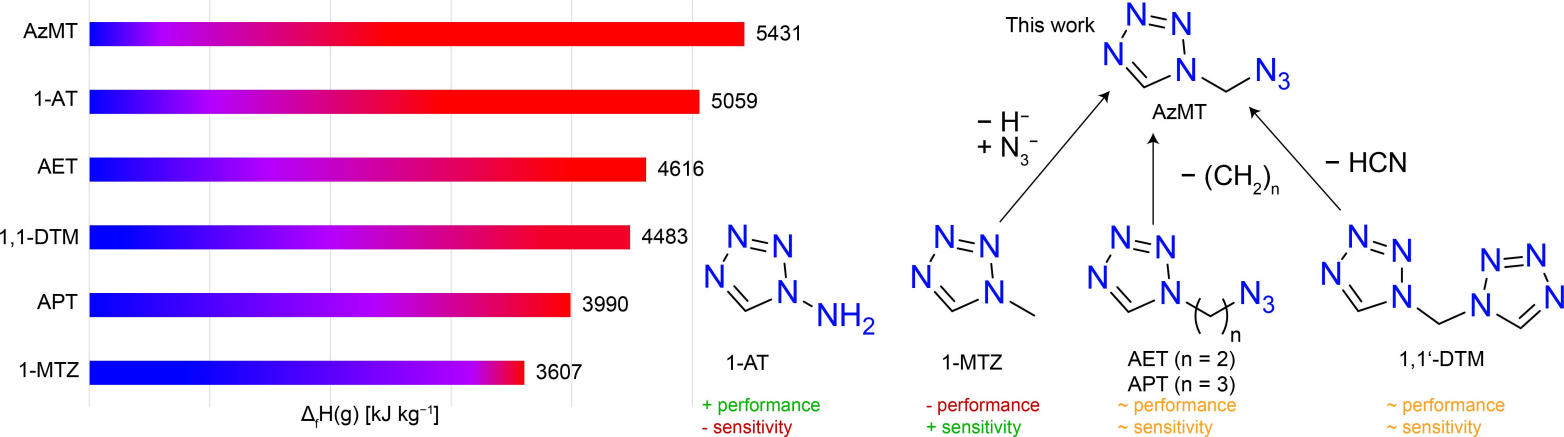
Molecular structures of 1‐methyl‐5*H*‐tetrazole (1‐MTZ), di(tetrazol‐1‐yl)‐methan (1,1’‐DTM), 1‐(azidopropyl)‐5*H*‐tetrazole (APT), 1‐(azidoethyl)‐5*H*‐tetrazole (AET), 1‐amino‐5*H*‐tetrazole (1‐AT) and 1‐(azidomethyl)‐5*H*‐tetrazole (AzMT). The structural relationship between 1‐MTZ, 1,1‐DTM and AET/APT is indicated (right), and a comparison of the calculated gas‐phase enthalpies of formation of all six ligands is shown on the left.

Another promising ligand is di‐(tetrazol‐1,1’‐yl)‐methane (1,1’‐DTM), being structurally similar to 1‐MTZ, having one proton substituted by another tetrazole moiety (Figure 1). 1,1’‐DTM validated the concept of adjustability of energetic coordination compounds by selecting appropriate metal cations and energetic anions, as can be seen from the different sensitivities of the copper(II) perchlorate (<1 *J*, <1 N) and the copper(II) picrate (6 *J*, >360 N) coordination compounds.

Considering the molecular structures of 1‐MTZ, AET, and 1,1’‐DTM, a structurally similar, yet missing compound is 1‐(azidomethyl)‐5*H*‐tetrazole. It is formally obtained by substitution of one hydrogen atom of 1‐MTZ by an azide functionality, as well as formally removing hydrogen cyanide from 1,1’‐DTM (Figure 1). More obvious, it can be obtained by formally further decreasing the alkyl chain from AET to a methylene moiety. Independent from the synthesis, the resulting 1‐(azidomethyl)‐5*H*‐tetrazol represents the smallest tetrazole with an *N*‐azidoalkyl functionality, having a drastically increased gas‐phase enthalpy of formation (5431 kJ kg^−1^), compared to its three parent compounds (Figure 1), even surpassing 1‐AT. Although methylene bridged functional groups on the tetrazole moiety can be found in literature,[^24^, ^25^, ^26^] especially 5‐(azidomethyl)‐1*H*‐tetrazole,^[27]^ they always show 5‐substitution of the tetrazole ring.[^28^, ^29^] Sadly, all 5‐substituted derivatives still exhibit acidic protons, making them unsuitable for use as ligands in energetic coordination compounds.

Independent of the synthesis, the resulting 1‐(azidomethyl)‐5*H*‐tetrazol represents the smallest tetrazole with an *N*‐azidoalkyl functionality, having a drastically increased gas‐phase enthalpy of formation (5431 kJ kg^−1^), compared to its three parent compounds (Figure 1), only surpassed by 1‐AT. Therefore, we opted to synthesize the highly energetic and hardly accessible 1‐(azidomethyl)‐5*H*‐tetrazole (**3**) for the first time. Here, next to the synthesis and characterization of highly energetic 1‐(azidomethyl)‐5*H*‐tetrazole, several energetic coordination compounds of the new ligand were synthesized. Single‐crystal X‐ray diffraction experiments were conducted, and all compounds were analyzed by elemental analysis and IR spectroscopy. The ligand was characterized by multinuclear NMR (^1^H, ^13^C, ^14^N, and ^15^N) spectroscopy, and the thermal and physicochemical properties of all compounds were determined.

## Results and Discussion

### Synthesis

***CAUTION***! The synthetic work described in this section involves the handling of very sensitive intermediates such as 1‐(azidomethyl)‐5*H*‐tetrazole (**3**) and ECCs **5**–**14**. Proper protective measurements and equipment must be used!

**Scheme 1:**
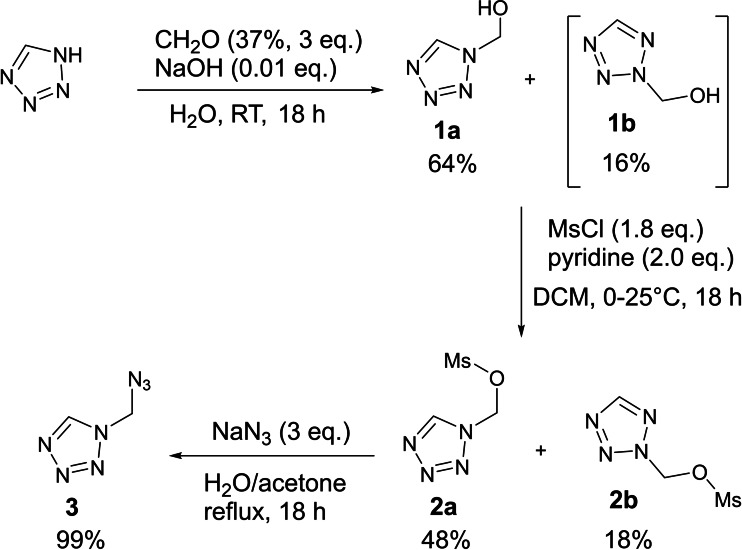
Synthesis of 1‐(azidomethyl)‐5*H*‐tetrazole (AzMT, **3**), starting from 1,5*H*‐tetrazole.

The synthesis of 1‐(azidomethyl)‐5*H*‐tetrazol (**3**) is shown in Scheme 1. The known literature^[30]^ hydroxy methylation of 1,5*H*‐tetrazole with formaldehyde (37 %) in aqueous solution was optimized towards a 66 % shorter reaction time with an improved yield of 80 % (over 65 %). An isomeric mixture of 1‐ and 2‐(hydroxymethyl)‐5*H*‐tetrazole (**1 a**, **1 b**) was obtained as a colorless oil. Due to the polarity of the alcohol functionality, a separation of the isomers by column chromatography was not possible at this stage. The isomeric mixture **1** was mesylated by methanesulfonyl chloride in CH_2_Cl_2_ at room temperature with a good overall yield of 64 %. After the reaction solution had been quenched in water, pure 1‐(methanesulfonyl‐methyl)‐5*H*‐tetrazole (**2 a**) precipitated and was filtered off. Compound **2 a** was obtained as a colorless solid in moderate yield (48 %). The organic layer was separated, washed with hydrochloric acid (2 m) and water, and the solvent was removed in vacuo to obtain crude 2‐(methanesulfonyl‐methyl)‐5*H*‐tetrazole (**2 b**) in poor yield (18 %). After refluxing **2 a** with an excess of sodium azide in a water/acetone mixture (1 : 1) overnight, the reaction solution was extracted with CH_2_Cl_2_ and the organic‐phase was washed with water and dried over magnesium sulfate. By removing the solvent in vacuo, 1‐(azidomethyl)‐5*H*‐tetrazole (**3**) was obtained as colorless solid in quantitative yield. Crystals suitable for single‐crystal X‐ray diffraction experiments were obtained by recrystallization from acetone.

### NMR spectroscopy

The ^1^H NMR of the crude isomeric mixture **1** in [D_6_]DMSO shows two signals at *δ*=9.31 and 8.77 ppm attributed to the tetrazole protons of **1 a** and **1 b** isomers, respectively. Integration of the signals reveals a distribution of 4/1 favoring **1 a** over **1 b**. The two signals at *δ*=6.11 and 6.03 ppm are attributed to the respective CH_2_ group of **1 a** and **1 b**. The ^1^H NMR of **2 a** in [D_6_]acetone shows three signals at *δ*=9.43, 6.67, and 3.27 ppm attributed to the CH, the CH_2_ and the CH_3_ group, respectively. The ^1^H NMR of compound **3** in [D_6_]DMSO shows two signals at *δ*=9.60 and 6.02 ppm attributed to the CH and CH_2_ group. Compared to **1 a**, the tetrazole CH is downfield shifted by 0.29 ppm whereas the upfield shift of the CH_2_ unit (0.01 ppm) is negligible. The ^13^C NMR spectrum also shows two signals at *δ*=144.2 and 60.5 ppm, marking the tetrazole and the methylene carbon atoms, respectively. ^14^N NMR measurement of **3** in [D_6_]DMSO shows two broadened signals at *δ*=−135.6 and −160.4 ppm corresponding to the N_β_ atom of the azide moiety and to the N_γ_ atom of the tetrazole.

**Figure 2:**
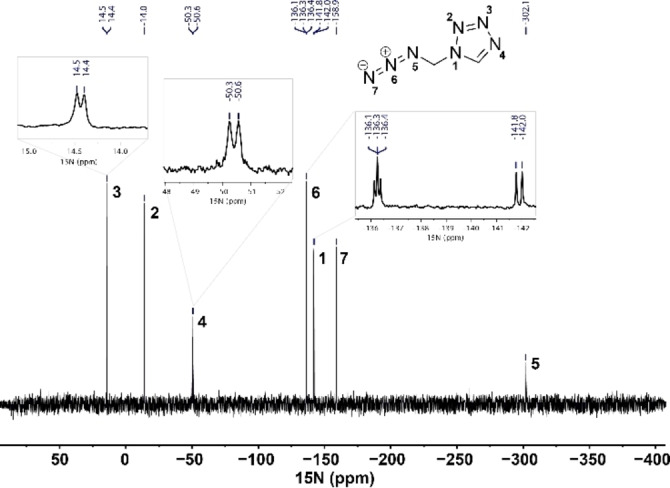
^15^N NMR spectrum of 1‐(azidomethyl)‐5*H*‐tetrazole (**3**) in [D_6_]acetone.

Due to the invisibility of the missing nitrogen atoms, proton coupled ^15^N NMR measurement (Figure 2) was performed. The resonances for the azide at *δ*=−302.1 (N_α_), −158.9 (N_γ_), and −136.3 (N_β_) are in the same range as comparable azido‐methyl compounds. The signal at *δ*=−136.3 splits into a triplet due to ^3^ *J* coupling to the methylene protons. The signals for the tetrazole at *δ*=14.4 (N_3_), −14.0 (N_2_), −50.4 (N_4_), and −141.9 ppm (N_1_) are also in the typical range of 1 *N*‐subsituted tetrazoles.^[22]^ Whereas the signal at *δ*=−14.0 (N_2_) appears as a singlet, the remaining resonances appear as doublets with coupling constants of 3.3 Hz (14.4 ppm), 12.7 Hz (−50.4 ppm), and 9.5 Hz (−50.4 ppm) due to ^3^ *J* and ^2^ *J* coupling with the tetrazole proton.

### Detonation properties

The detonation performance parameters of **3** were calculated with the explo5 program code,^[31]^ for which the enthalpy of formation was determined by applying the atomization method using room temperature CBS‐4M enthalpy. As the performance properties for AET and APT are not reported in literature, their enthalpies of formation were also calculated to further obtain their detonation performances. Table 1 compares all calculated values for **3**, AET and APT. A small increase in density of 0.06 g cm^−3^ (Table 1) is observed by decreasing the alkyl‐chain by one CH_2_ group from APT to AET, whereas further shortening the alkyl chain by a second CH_2_ group to AzMT causes a more drastic increase in density by 0.24 g cm^−3^. The higher increase in density can be explained due to the liquid state of APT and AET at room temperature, whereas AzMT is already a solid at room temperature. Additionally, the enthalpy of formation from **3** (654.5 kJ mol^−1^) to APT (568.4 kJ mol^−1^) also decreases drastically. As the performance of energetic compounds depends on their enthalpy of formation and their densities, combining both into an enthalpy density offers an appropriate value to quickly elucidate the differences. Here, AzMT shows a very high enthalpy density of 8110 J cm^−3^, followed by AET (5630 J cm^−3^) and APT (4639 J cm^−3^). Therefore, the calculated detonation performances show a drastic increase in performance from APT (6757 m s^−1^) to AzMT (8124 m s^−1^), hence a significantly higher performance of the corresponding ECCs can also be estimated.

**Table 1:** Energetic properties of AzMT (**3**), compared to AET^[23]^ and APT.^[22]^

	AzMT (**3**)	AET^[23]^	APT^[22]^
Formula	C_2_H_3_N_7_	C_3_H_5_N_7_	C_4_H_7_N_7_
*M* [g mol^−1^]	125.1	139.1	153.2
*ρ* [g cm^−3^]	1.545^[a]^	1.31^[b]^	1.25^[b]^
*N* [%]^[c]^	78.38	70.48	64.02
*Ω_CO_* [%]^[d]^	−70.34	−97.75	−120.13
Δ_*f*_ *H°* [kJ mol^−1^]^[e]^	655	598	568
Δ_*f*_ *H°* [kJ kg^−1^]^[f]^	5232	4427	3853
Δ_*f*_ *H°* [J cm^−3^] ^[g]^	8110	5630	4639
explo5 V6.05.04			
−Δ_*ex*_ *U* ^0^ [kJ kg^−1^]^[h]^	5127	4449	4121
*T*_det_ [K]^[i]^	3473	3169	2609
*V*_0_ [L kg^−1^]^[j]^	502	540	620
*P*_CJ_ [kbar]^[k]^	229	192	130
*V*_det_ [m s^−1^]^[l]^	8124	7086	6757

[a] From single‐crystal X‐ray diffraction analysis recalculated to room temperature. [b] Measured with a gas pycnometer. [c] Nitrogen content. [d] Oxygen balance towards the formation of CO. [e] Calculated enthalpy of formation. [f] Calculated mass related enthalpy of formation. [g] Calculated volume related enthalpy of formation. [h] Energy of explosion. [i] Detonation temperature. [j] Volume of detonation products (assuming only gaseous products). [k] Detonation pressure at Chapman‐Jouguet point. [l] Detonation velocity.

### Energetic coordination compounds

**Scheme 2:**
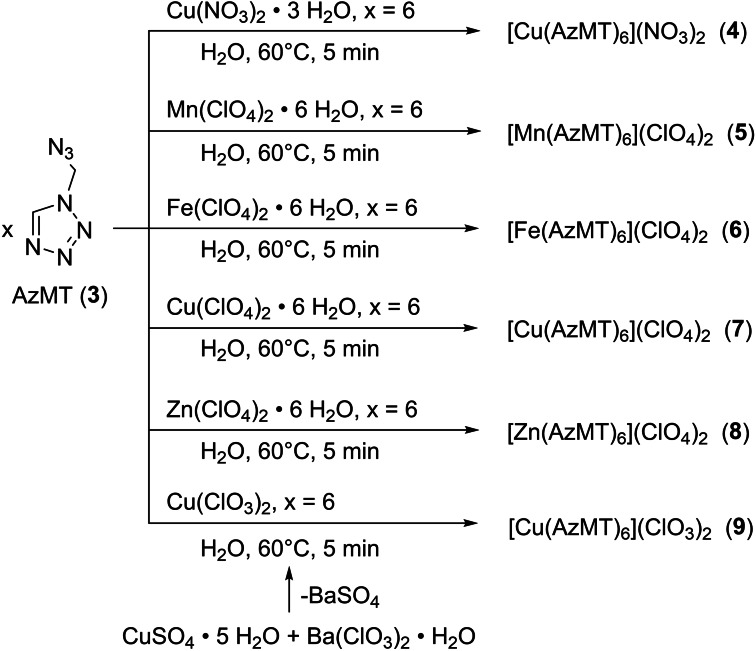
Synthesis of energetic coordination compounds of copper(II) nitrate (**4**), 3d‐metal perchlorates (**5**–**8**) and copper(II) chlorate (**9**) with 1‐(azidomethyl)‐5*H*‐tetrazole.

Due to the absence of acidic protons in compound **3**, it can be used as a well‐suited neutral ligand for ECCs. The application of several oxidizing anions such as nitrate, chlorate, perchlorate, fulminate, picrate, styphnate, and trinitro phloroglucinol, and reducing anions such as azide, enables the tuning of the energetic properties of the resulting coordination compound. The energetic coordination compounds **4**–**9** were obtained by dissolving the corresponding metal(II) salts in water at elevated temperatures (60–80 °C) and adding **3** in stoichiometric amounts (Scheme 2). Due to the commercial unavailability of copper(II) chlorate, it was synthesized according to a previously published procedure.^[23]^

**Scheme 3:**
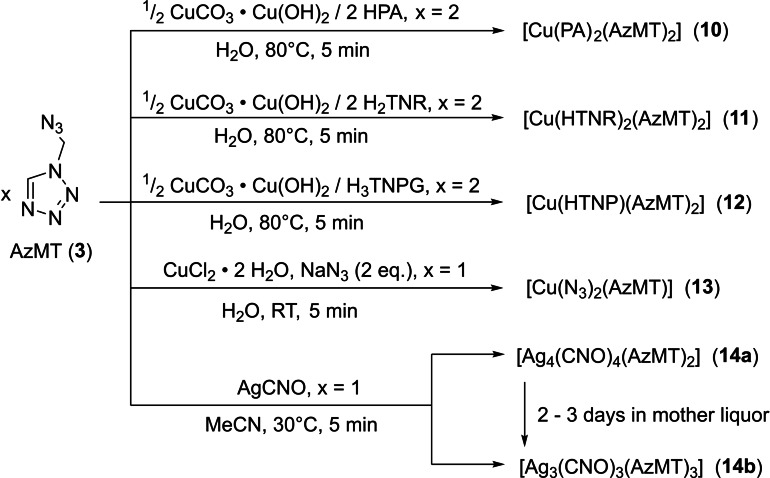
Synthesis of nitroaromatic coordination compounds **10**–**12**, as well as the coordination compounds of copper(II) azide (**13**) and silver(I) fulminate (**14 a/b**) with AzMT.

The copper(II) nitroaromatic salts for compounds **10**–**12** were synthesized by dissolving basic copper(II) carbonate in water and adding stoichiometric amounts of the free acids of the corresponding nitroaromatic anion (Scheme 3). Compound **13** was synthesized by dissolving copper(II) chloride dihydrate in water, adding one equivalent of **3**, followed by adding two equivalents of sodium azide dissolved in water. Immediately after the addition of the azide, a brown precipitate of **13** formed, was filtered off and washed with little hot water.

Compound **14 a/b** was obtained by dissolving silver(I) fulminate in acetonitrile at 30 °C and adding one equivalent of ligand. Crystals of compound **14 a** start to form in the mother liquor after one day. By filtering off the platelets, crystals suitable for single‐crystal X‐ray diffraction are obtained. When leaving the mother liquor for several days, white needles, suitable for X‐ray diffraction, of compound **14 b** start to crystallize on top of the crystals of **14 a**, ultimately completely turning **14 a** into **14 b**. Crystallization of the coordination compounds **4**–**12** was achieved by slowly evaporating the mother liquor for 1–2 days, and collecting the formed solids by filtration with subsequent drying in air. Single crystals suitable for X‐ray diffraction experiments were also obtained from the mother liquor for compounds **4**, **9**, and **10**. Single crystals of compound **13** were obtained by a three‐layered crystallization approach. Sodium azide together with **3** was dissolved in water and added to a test tube. A second layer consisting of a 1 : 1 mixture of water and ethanol was layered on top, and the third layer of copper(II) chloride dissolved in ethanol was slowly added. The test tube was carefully stoppered as not to mix the three layers and was set aside. After several days, crystals of compound **13** had formed and were collected by filtration and washed with little hot water. It was not possible to obtain crystals suitable for single‐crystal X‐ray diffraction of compounds **5**–**8**, **11**, and **12**; their composition was analyzed and proven by elemental analysis. Generally, all energetic coordination compounds were fully characterized by elemental analysis, infrared spectroscopy, differential thermal analysis, as well as their sensitivity towards impact (IS), friction (FS), and electrostatic discharge (ESD) were tested. Hot needle (HN) and hot plate (HP) tests were performed to obtain insight into their initiation capabilities, which were then tested for several chosen compounds.

### Crystal structures

Solid‐state crystal structures of compounds **3**, **4**, **9**, **10**, and **14 a/b** were determined by using low‐temperature single‐crystal X‐ray diffraction. All of the data and parameters of the measurements as well as of the refinements are given in Table S1 in the Supporting Information. All crystal densities were recalculated to their respective room temperature crystal density.

Deposition Numbers 2124092 (for **3**), 2124093 (for **10**), 2124094 (for **9**), 2124095 (for **13**), 2124096 (for **14 b**), 2124097 (for **4**), 2144883 (for **14 a**) contain the supplementary crystallographic data for this paper. These data are provided free of charge by the joint Cambridge Crystallographic Data Centre and Fachinformationszentrum Karlsruhe Access Structures service.

**Figure 3:**
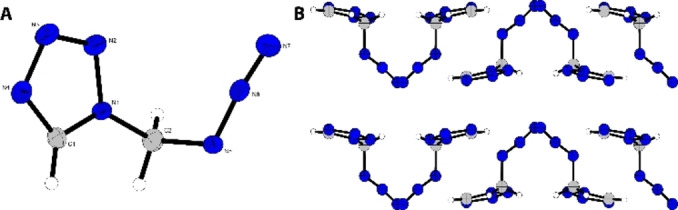
A) Crystal structure of 1‐(azidomethyl)‐5*H*‐tetrazole. B) Crystal packing along the *a*‐axis; ellipsoids in all structures are shown with a probability of 50 %; Selected bond lengths [Å]: N1‐N2 1.35(2), N1‐C1 1.33(3), N1‐C2 1.46(3), N2‐N3 1.29(3), N3‐N4 1.37(3), N4‐C1 1.31(3), N5‐C2 1.47(4), N5‐N6 1.25(3), N6‐N7 1.12(3); angles [°]: N2‐N1‐C1 108.0(15), N1‐C2‐N5 111.6(2), N2‐N1‐C2 120.8(18), N1‐C1‐H1 125.3(13), C1‐N1‐C2 131.2(18), N4‐C1‐H1 124.8(13), N1‐N2‐N3 106.1(17), N1‐C2‐H2 A 107.9(12), N2‐N3‐N4 111.0(18), N1‐C2‐H2B 106.1(13), N3‐N4‐C1 105.0(19), N5‐C2‐H2 A 112.4(13), N6‐N5‐C2 113.9(19), N5‐C2‐H2B 107.3(13), N5‐N6‐N7 172.6(3), H2A‐C2‐H2B 111.4(18), N1‐C1‐N4 109.9(19).

Compound **3** crystallizes in the orthorhombic space group *Pbca* with eight formula units in the unit cell and a density of 1.55 g cm^−3^ (Figure 3). All bond lengths are in the typical range for C−N, N−N single and double bonds. The tetrazole moiety forms a nearly perfectly level plane (C1‐N1‐N2‐N3=0.3°) from which the methylene group protrudes slightly by an angle of C1‐N1‐N2‐N3=1.4°. Typical for covalently bound azides, the −N_3_ unit protrudes from the tetrazole plane with an angle of N1‐C2‐N5=111° as shown in Figure 3.

**Figure 4:**
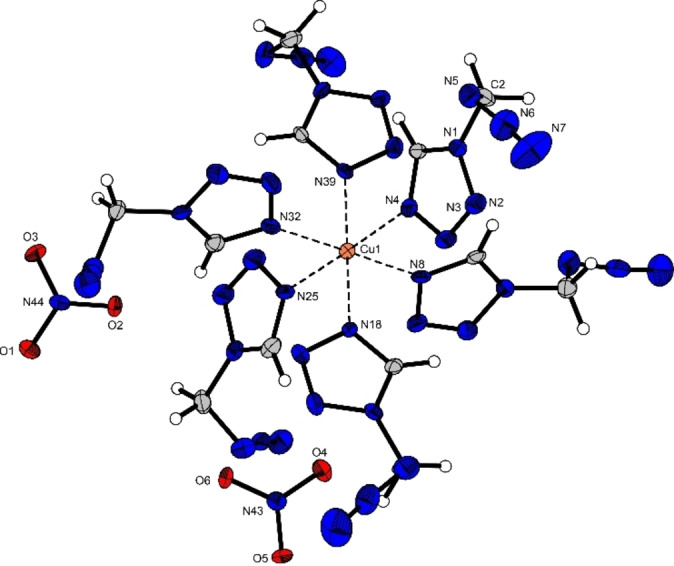
Molecular unit of [Cu(AzMT)_6_](NO_3_)_2_ (**4**); Selected bond lengths [Å]: Cu1‐N4 2.30(6), Cu1‐N8 2.07(6), Cu1‐N18 2.02(6), Cu1‐N25 2.33(6), Cu1‐N32 2.07(6), Cu1‐N39 2.03(6); angles [°]: N4‐Cu1‐N8 89.5(2), N4‐Cu1‐N18 91.6(2), N4‐Cu1‐N25 178.3(2), N4‐Cu1‐N32 93.5(2), N4‐Cu1‐N39 87.9(2).

Compound **4** crystallizes in the monoclinic space group *Cc* with four formula units in the unit cell and a density of 1.66 g cm^−3^ (Figure 4). The copper(II) cation is sixfold coordinated by 1‐(azidomethyl)‐5*H*‐tetrazole ligands whereas the two nitrate anions are non‐coordinating. While the bond lengths within the six ligands are equivalent as for pure compound **3**, the bond lengths towards the copper(II) center range between 2.02–2.33 Å with angles of nearly perfect 180° between opposing ligands. Two of them (N4 and N25) show a longer bond distance towards the copper(II) cation than the four other ligands. Hence, the copper(II) cation is coordinated in a square bipyramidal conformation, caused by a typical Jahn–Teller‐like distortion, due to the electron configuration of [Ar]3d^9^ of the copper(II) cation.

**Figure 5:**
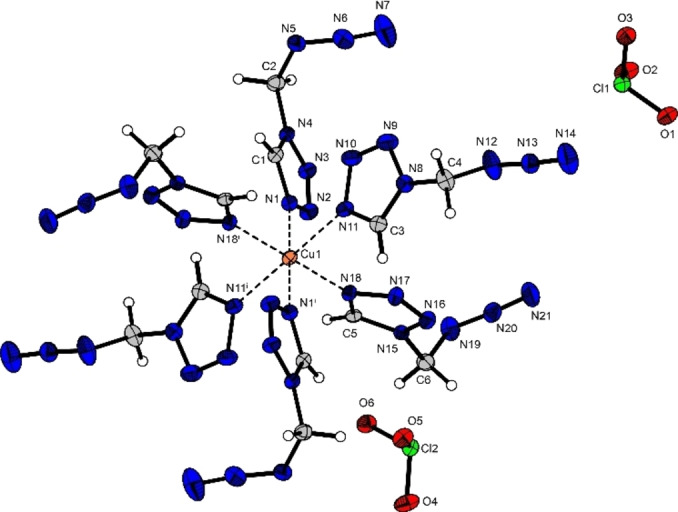
Molecular unit of [Cu(AzMT)_6_](ClO_3_)_2_ (**9**); Selected bond lengths [Å]: Cu1‐N1 2.05(2), Cu1‐N11 2.36(2), Cu1‐N18 2.02(2); angles [°]: N1‐Cu1‐N11 91.2(9), N1‐Cu1‐N18 91.6(8), N11‐Cu1‐N11^i^ 180.0.

The chlorate complex **9** crystallizes in the triclinic space group *P* $\bar{1}$ with two formula units in the unit cell and a density of 1.71 g cm^−3^ (Figure 5). The copper(II) cation is sixfold coordinated by AzMT ligands, all binding via their respective N4‐nitrogen atom. Like in **4**, the coordination sphere is a square bipyramid due to Jahn–Teller‐like distortion along the N1‐Cu1‐N1^i^ axis. The change of anion from nitrate (**4**) to chlorate (**9**) does not induce a change of bond lengths within the ligands. The bond lengths between the ligands and the copper(II) center are in the same range as in **4** (2.02–2.36 Å). The two chlorate anions are also non‐coordinating.

**Figure 6:**
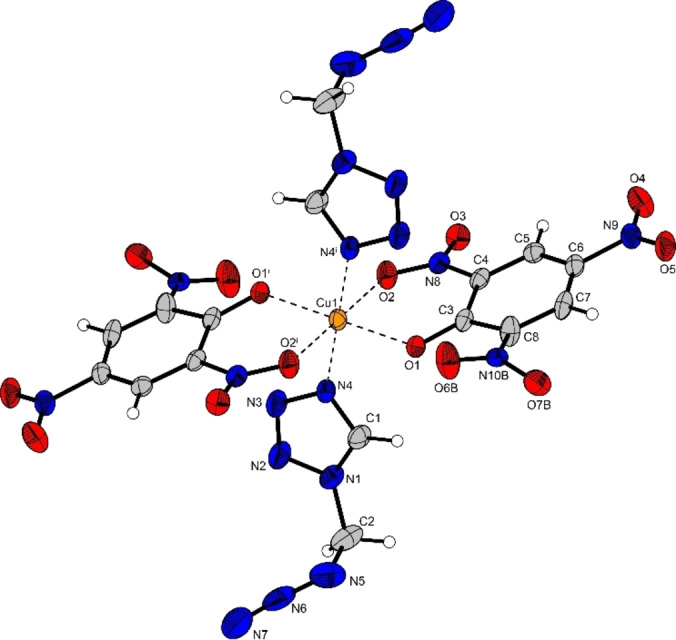
Molecular unit of [Cu(PA)_2_(AzMT)_2_] (**10**); Selected bond lengths [Å]: Cu1‐O1 1.92(2), Cu1‐O2 2.35(2), Cu1‐N4 2.01(2); angles [°]: O1‐Cu1‐O2 78.9(7), O1‐Cu1‐N4 86.8(7), O2‐Cu1‐N4 90.13(7).

Copper(II) picrate **10** (Figure 6) crystallizes in the monoclinic space group *P*2_1_/*c* with two formula units in the unit cell and a density of 1.83 g cm^−3^. The copper(II) cation is coordinated by two equatorial AzMT ligands and two coordinating picrate anions, acting as bidentate ligands. The two picrates coordinate via their deprotonated hydroxy group as well as a second oxygen atom of one of the *ortho*‐ nitro groups. The bond lengths between the copper(II) and the AzMT ligands (2.01 Å) are in the same range as for the copper(II) nitrate (**4**) and chlorate (**9**). The deprotonated hydroxy groups also coordinate in the equatorial plane, whereas the nitro groups oxygen atoms are axial standing with a bond length of 2.35 Å, resulting in a typical Jahn‐Teller‐like distortion along the O2‐Cu1‐O2^i^ axis.

**Figure 7:**
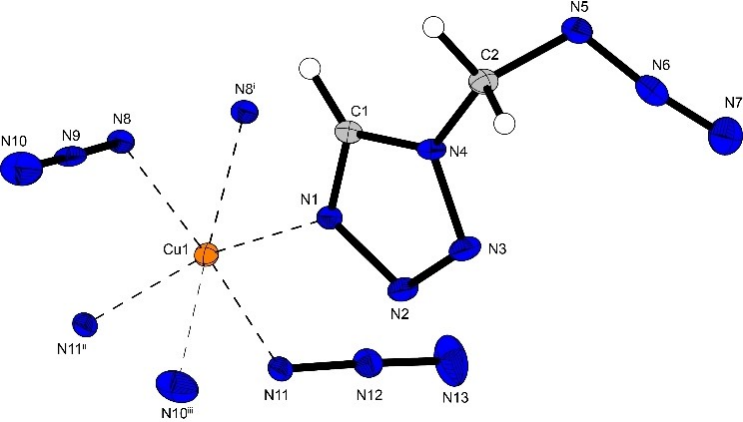
Molecular unit of [Cu(N_3_)_2_(AzMT)] (**13**); Selected bond lengths [Å]: Cu1‐N1 2.01(2), Cu1‐N8 1.97(2), Cu1‐N11 2.00(2), Cu1‐N10^iii^ 2.65(3), Cu1‐N11^ii^ 2.02(2), Cu1‐N8^i^ 2.43(2); angles [°]: N1‐Cu1‐N11^ii^ 166.5(9), N8‐Cu1‐N11 172.8(9), N8^i^‐Cu1‐N10^iii^ 169.4(8), N1‐Cu1‐N8 91.7(9), N1‐Cu1‐N11 95.2(9).

The copper(II) azide complex **13** crystallizes in the monoclinic space group *P*2_1_/*c* with four formula units in the unit cell and a density of 1.93 g cm^−3^. The copper(II) cation is coordinated by five azide anions as well as one AzMT (**3**) ligand (Figure 7) forming a strongly distorted octahedron due to high deviations from the 90° coordination angles, observed in compounds **4**, **9**, and **10**. The deviation of up to 13.5° (N1‐Cu1‐N11^ii^) from a linear arrangement of two opposing coordinating compounds is caused by two different bridging modes of the azide anions. Two of the coordinating azide anions bridge between two copper cations, thereby forming strands that are interconnected by the other three anions, which bridge between three copper cations.

**Figure 8:**
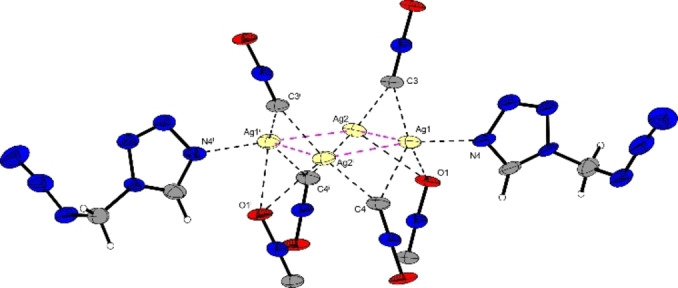
Molecular unit of [Ag_4_(CNO)_4_(AzMT)_2_] (**14 a**); Selected bond lengths [Å]: Ag1‐Ag2 2.91(8), Ag1‐Ag2^i^ 2.77(7), Ag1‐N4 2.29(5), Ag1‐C3 2.27(5), Ag1‐O2 2.66(3), Ag2‐C3 2.17(5); angles [°]: Ag2‐Ag1‐N4 121.92(12), N4‐Ag1‐C3 100.34(18), Ag2‐C3‐Ag1 81.94(20), Ag2‐Ag1‐O2 129.02(11), Ag2^i^‐Ag1‐O2 66.85(10), Ag1‐Ag2^i^‐C3^i^ 93.11(13), Ag2^i^‐Ag1‐C3 74.66(11); Torsion angles [°] Ag2^i^‐Ag1‐Ag2‐Ag1^i^ 3.32(2).

Interestingly, the coordination of the copper(II) cation, as well as the two different bridging modes of the azide anions, are nearly identical to those of the 1‐MTZ analogous, proving the structural similarity of the 1‐MTZ and AzMT ligand.^[32]^ For the silver(I) fulminate complexes, crystallization in two different space groups was observed. The initially formed platelets (**14 a**) crystallized in the monoclinic space group *I*2/*a* with eight formula units in the unit cell and a density of 2.71 g cm^−3^, the highest of all herein investigated compounds (Figure 8). The molecular unit consists of a tetrameric cluster of silver fulminate (SF), with the silver cations forming a nearly planar rhombus with angles of 75.72° (Ag2^i^‐Ag1‐Ag2) and 104.28° (Ag1‐Ag2‐Ag1^i^), and a torsion angle of 3.32°. The fulminate anions bridge between the silver cations with their terminal carbon and oxygen atoms, having shorter Ag‐C (2.17–2.27 Å) interactions than Ag−O interactions (2.50–2.73 Å). Like in previously reported silver fulminate complexes,^[33]^ the carbon atoms of the fulminate anions bridge between two silver cations of a tetramer. While the same bridging between two silver cations of the tetramer is observed for the oxygen atoms, a third interaction with a silver cation of a second tetrameric unit causes the formation of an extended three‐dimensional structure. Due to the bridging character of the fulminate anions, Ag−Ag interactions (Figure 8) are observed within the tetrameric unit. Comparable to previously reported coordination compounds of SF with monodentate ligands,^[33]^ the bond lengths of these argentophilic interactions (2.91–2.77 Å) are in the range to be classified as sub‐van der Waals contacts (<3.44 Å).^[34]^ Additionally, the shortest argentophilic interaction observed has a distance of 2.7675(7) Å, being close to the shortest reported distance of 2.746(1) Å.^[34]^

**Figure 9:**
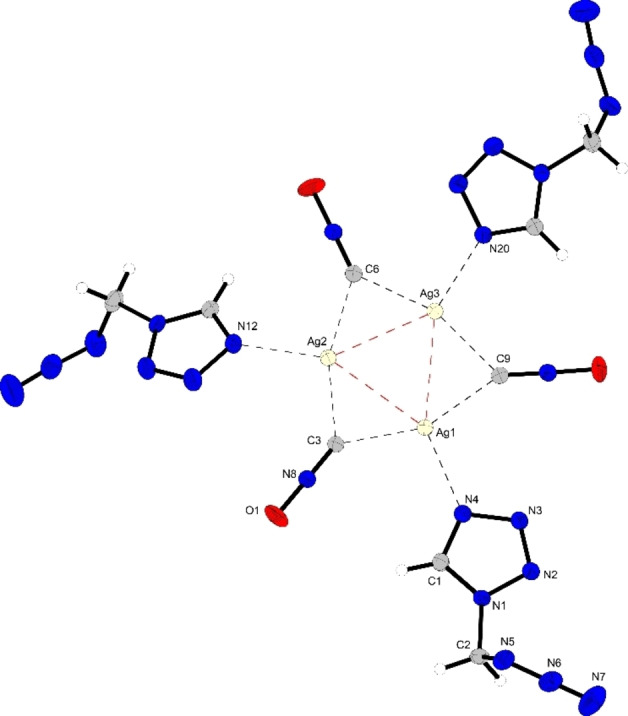
Molecular unit of [Ag_3_(CNO)_3_(AzMT)_3_] (**14 b**); Selected bond lengths [Å]: Ag1‐Ag2 2.87(7), Ag1‐Ag3 2.84(7), Ag2‐Ag3 2.81(7), Ag1‐N4 2.31(2), Ag2‐N12 2.31(2), Ag3‐N20 2.31(2), Ag1‐C3 2.22(3), Ag1‐C9 2.20(2), Ag2‐C3 2.17(2), Ag2‐C6 2.19(2), Ag3‐C6 2.18(3), Ag3‐C9 2.20(3); angles [°]: Ag1‐Ag2‐Ag3 59.9(1), Ag2‐Ag1‐Ag3 58.9(1), Ag1‐Ag3‐Ag2 61.2(1), Ag1‐C3‐Ag2 81.7(8), Ag2‐C6‐Ag3 79.7(9), Ag1‐C9‐Ag3 80.4(9), C3‐Ag1‐C9 155.2(9), C3‐Ag2‐C6 159.1(10), C6‐Ag3‐C9 159.3(9).

After the mother liquor had stood for several days, white crystalline needles started to form on top of the platelets. These crystalline needles were also suitable for single‐crystal X‐ray diffraction experiments, showing compound **14 b** crystallizing in the triclinic space group *P* $\bar{1}$ with two formula units in the unit cell and a density of 2.30 g cm^−3^. In contrast to **14 a**, the molecular unit consists of a trimeric cluster of silver fulminate coordinated by three AzMT ligands (Figure 9). Here, the fulminate anions also bridge between silver cations, but in contrast to **14 a**, only by their terminal carbon atom, forming monomers with argentophilic interactions between the silver cations, slightly longer (2.81–2.87 Å) than those in the tetrameric structure.

As in the tetrameric structure, the silver fulminate cluster is nearly planar with a torsion angle of 7.8° (C6‐Ag2‐Ag1‐C9). Interestingly, a different protrusion for every AzMT ligand from the plane, formed by the SF trimer, is observed. While the first AzMT (via N4) is nearly perpendicular with the plane, the AzMT ligands (coordinated via N12 and N20) protrude from the plane by 8.0° and 46.5°, respectively.

### Physicochemical properties

#### Thermal behavior

**Figure 10:**
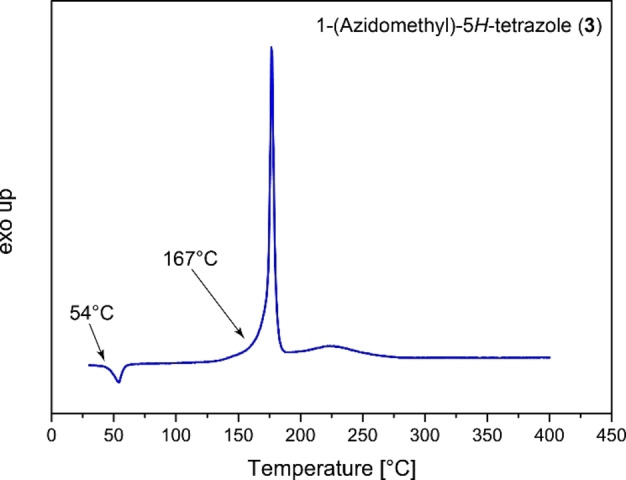
Differential thermal analysis of compound **3** with a heating rate of 5 °C min^−1^.

**Figure 11:**
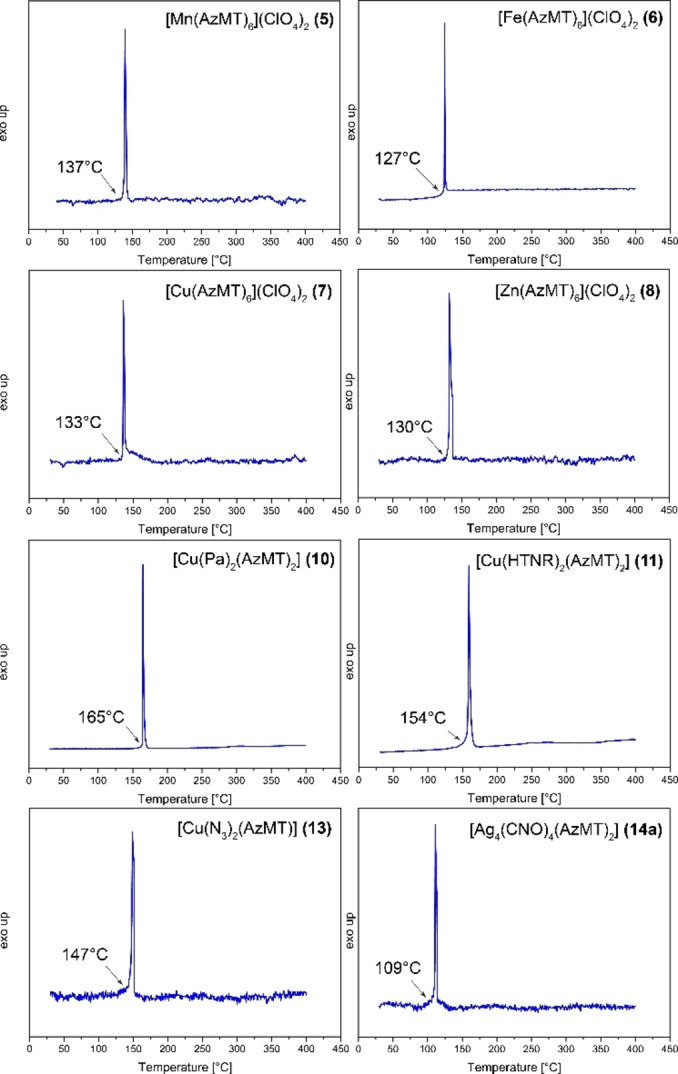
Differential thermal analysis of compounds **5**–**8**, **10**–**11**, and **13**–**14 a**, with a heating rate of 5 °C min^−1^.

Differential thermal analyses of compounds **3**–**14** are shown in Figures 10 and 11. DTA plots for compounds **4**, **9** and **12** are shown in the Supporting Information. Compound **3** shows an endothermic event at 54 °C, due to a melting of the compound, and an exothermic decomposition temperature of 167 °C, lower than the comparable 1‐(azidoethyl)‐5*H*‐tetrazole (AET) derivative (193 °C).^[23]^ All energetic coordination compounds show lower thermal stability, which is also observed for ECCs of AET. Compound **4** shows a small endothermic event at 60 °C, attributing a melting of the compound, with a first exothermic event happening already at 123 °C contributing to explosive decomposition. The same behavior is observed for compound **9**, showing an endothermic event at 112 °C, immediately evolving into an exothermic event at 123 °C. The metal perchlorate compounds **5**–**8** exhibit exothermic decompositions between 127 (**6**) and 137 °C (**5**) where all four compounds violently detonate. The nitroaromatic compounds **10** (165 °C), **11** (154 °C), and **12** (151 °C) exhibit slightly higher thermal stabilities, yet they are less stable than pure **3**. The coordination compounds of copper(II) azide (**13**) and silver(I) fulminate (**14 b**) are thermally stable up to 147 and 109 °C, respectively. Here the formation of complexes with 1‐(azidomethyl)‐5*H*‐tetrazole (**3**) leads to a more drastic drop in thermal stability compared to their parent salts copper(II) azide (205 °C) and silver(I) fulminate (196 °C).

#### Sensitivities

1‐(Azidomethyl)‐5*H*‐tetrazole (**3**) is a very sensitive compound with an impact and friction sensitivity of 2 J and 1 N (Table 2). (Both values are considered as very sensitive.) Although it is not sensitive (540 mJ) towards electrostatic discharge (ESD), due to its mechanical sensitivities, it has to be ranked as a primary explosive. By applying **3** as ligand in ECCs of copper(II) nitrate (**4**), picrate, (**10**), styphnate (**11**), and trinitrophloroglycinolate (**12**), it is possible to stabilize the friction sensitivity of **3**. While **4** is only slightly less sensitive (15 N) than pure **3**, compound **10** is completely insensitive (360 N). With an increasing amount of hydroxy groups, **11** shows a higher sensitivity (192 N) and **12** the highest friction sensitivity of 45 N. Yet all nitroaromatic compounds are far less sensitive than the copper(II) nitrate complex **4**. When changing from copper(II) nitrate to perchlorate (**7**) or chlorate (**9**) the resulting coordination compounds exhibits drastically increased sensitivities (<1 J, <0.1 N) compared to pure **3**. Changing to manganese(II) (**5**), iron(II) (**6**), or zinc(II) perchlorate (**8**) the same increase in sensitivities is observed. While all compounds show very high sensitivities towards impact (<1 J), compound **5** towards friction (0.5 N) than **6**, **7**, and **8** (<0.1 N). Compound **13** also shows a very high mechanical sensitivity of <1 J and <0.1 N. Yet it seems to be more stable than pure copper(II) azide, derived from the fact that we had no problems handling compound **13**, while several minor explosions happened when handling pure copper(II) azide. The coordination compound of silver(I) fulminate with **3** leads to an increase in impact sensitivity (<1 J) compared to pure silver fulminate (5 J), whereas the friction sensitivity is approximately equal to pure SF (<0.1 N). The same trends of stabilization or destabilization as for the mechanical sensitivities of the ligand (**3**) is observed comparing the ESD sensitivities. Compounds **4**, **10**, and **11** are completely insensitive towards ESD (>1500 mJ), while compound **12** (1220 mJ) is nearly insensitive. Changing to metal perchlorates or chlorates the ESD sensitivity of the resulting complex (**6**: 380 mJ; **7**: 480 mJ; **8**: 380 mJ; **9**: 380 mJ) is increased compared to the pure ligand (540 mJ). Here, only compound **5**, the manganese perchlorate complex shows a slightly lower sensitivity (740 mJ) than **3**. The highest sensitivity towards ESD is observed for the silver(I) fulminate complex **14 b** (10 mJ), followed by the copper(II) azide complex **13** (38 mJ). Here, the addition of **3** to the corresponding salt results in a stabilization of the otherwise sensitive parent compound. Due to the high sensitivities of the metal (per‐)chlorates, as well as **14 b** towards impact (<1 J), ball drop impact measurements were performed, allowing a more detailed differentiation of the compounds. For compounds **5** and **7** (Table 1) a ball drop impact sensitivity (BDIS) of 25 mJ was observed, while compounds **8**, and **14 b** are slightly more sensitive (20 mJ) and iron(II) perchlorate (**6**), as well as the copper(II) chlorate (**9**) showed the highest sensitivity (16 mJ).

**Table 2:** Thermal stability^[a]^ as well as sensitivities to mechanical and electrical stimuli of compounds **3**–**14**, compared to copper azide and silver fulminate.

Compound	*T*_endo_^[b]^ [°C]	*T*_exo_^[c]^ [°C]	IS^[d]^ [J]	FS^[e]^ [N]	ESD^[f]^ [mJ]	BDIS^[g]^ [mJ]	HP	HN
AzMT (**3**)	54	167	2	1	540		Det	Def
[Cu(AzMT)_6_](NO_3_)_2_ (**4**)	60	123	2	15	>1500		Def	Def
[Mn(AzMT)_6_](ClO_4_)_2_ (**5**)		137	<1	0.5	740	25	Def	Det
[Fe(AzMT)_6_](ClO_4_)_2_ (**6**)		127	<1	<0.1	380	16	Det	Det
[Cu(AzMT)_6_](ClO_4_)_2_ (**7**)		133	<1	<0.1	480	25	Det	Det
[Zn(AzMT)_6_](ClO_4_)_2_ (**8**)		130	<1	<0.1	380	20	Def	Def
[Cu(AzMT)_6_](ClO_3_)_2_ (**9**)	112	123	<1	<0.1	380	16	Det	Def
[Cu(Pa)_2_(AzMT)_2_] (**10**)		165	<1	360	>1500		Def	Dec
[Cu(HTNR)_2_(AzMT)_2_] (**11**)		154	<1	192	>1500		Dec	Dec
[Cu(HTNPG)(AzMT)_2_] (**12**)		151	<1	45	1220		Def	Def
[Cu(N_3_)_2_(AzMT)] (**13**)		147	<1	<0.1	38		Det	Det
[Ag_4_(CNO)_4_(AzMT)_2_] (**14 a**)		109	<1	<0.1	10	20	Det	Det
Cu(N_3_)_2_		205	≪1	≪0.1	≤0.28			
AgCNO^[33]^		196	5	≤0.1	≤0.28

[a] DTA onset temperatures at a heating rate of 5 °C min^−1^. [b] Oset temperature of endothermic event in the DTA, indicating a melting of the compound. [c] Onset temperature of exothermic event in the DTA. [d] Impact sensitivity (BAM drop hammer (1 of 6)). [e] Friction sensitivity (BAM friction tester (1 of 6)). [f] Electrostatic discharge device (OZM XSpark10). [g] Ball drop impact sensitivity (OZM BIT‐132) determined with the 1‐of‐6 method in accordance with the MIL‐STD 1751 A (method 1016).

#### Initiation capabilities

**Figure 12:**
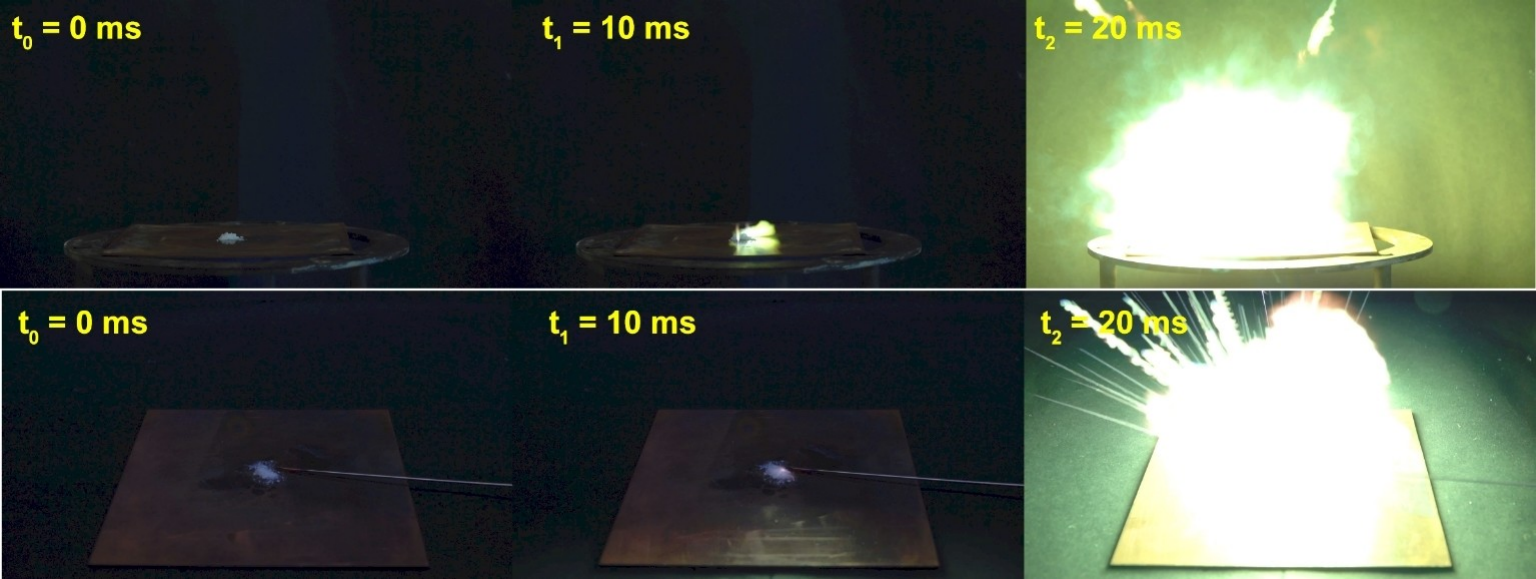
Top row: Hot‐plate test of [Mn(AzMT)_6_](ClO_4_)_2_ (**5**); Bottom row: Hot‐needle test of **5**; The time between each frame is 10 ms and shows the moment before (*t*_0_), at the onset of (*t*_1_) and during (*t*_2_) decomposition.

To get a further insight on the behavior upon fast heating with and without confinement, hot plate (HP) and hot needle (HN) tests were carried out. This also allows for a first evaluation of the applicability of the tested compounds as primary explosives. Detailed experimental procedures are given in the Supporting Information, the results are summarized in Table 1. Pure compound **3** showed a detonation in the HP test and a deflagration in the HN test. While the copper(II) nitrate (**4**) and the nitroaromatic compounds **10**, **11**, and **12** show deflagration or decomposition (**10**, **11**), the (per)chlorate metal complexes show a violent detonation (**6**, **7**, and **9**) or sharp deflagration (**5** and **8**) in the HP test (Figure 12). The complexes of copper(II) azide (**13**) and silver(I) fulminate (**14**) detonate both in the HP and HN test.

**Figure 13:**
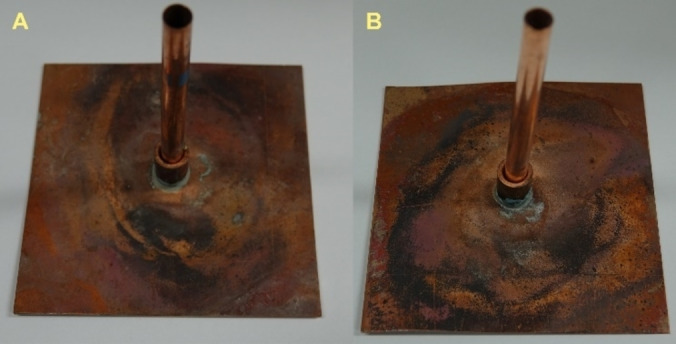
Intact witness plates and copper tubes after the initiation testing of compounds **3** and **12**.

**Figure 14:**
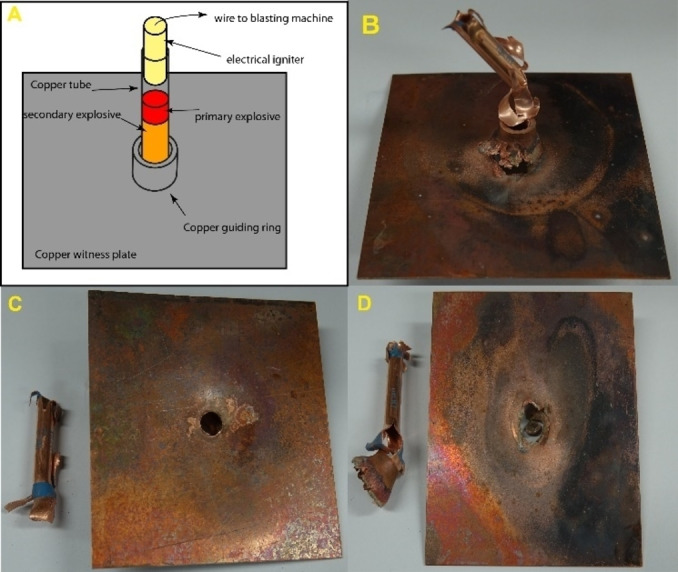
A) Schematic test setup for initiation testing. Positive initiation of PETN by B) [Mn(AzMT)_6_](ClO_4_)_2_ (**5**), C) [Cu(AzMT)_6_](ClO_4_)_2_ (**7**), and D) [Zn(AzMT)_6_](ClO_4_)_2_ (**8**), indicating a DDT for all three compounds. The positive initiation of PETN is indicated by the perforation of the copper witness plate.

For compounds **3**, **5**, **7**, **8**, and **12** initiation tests were performed. The detailed testing setup and procedure are given in the Supporting Information. Compounds **3** and **12** showed no positive initiation of PETN, which is indicated by the still intact witness plate (Figure 13). Therefore, neither compound was able to undergo deflagration to detonation transition (DDT) in this setup, which is a necessary characteristic for a compound to be used as a primary explosive. Nonetheless, the perchlorates **5**, **7**, and **8** were able to undergo DDT and initiate the PETN sample, indicated by a perforation of the copper witness plate (Figure 14).

## Conclusion

Successful hydroxymethylation of 1,5*H*‐tetrazole with aqueous formalin solution can only be achieved by the addition of catalytic amounts (3–5 wt %) of sodium hydroxide to the reaction solution. Subsequent protection of the alcohol by methanesulfonyl chloride provided the precursor compound needed to successfully synthesize the highly energetic 1‐(azidomethyl)‐5*H*‐tetrazole (**3**) for the first time. While its structural isomer 5‐(azidomethyl)‐1*H*‐tetrazole has already been reported, **3** represents the smallest tetrazole derivate with the shortest *N*‐alkyl‐azide functionality reported, showing a very high positive heat of formation. Single‐crystal X‐ray analysis of **3** was supplemented by elemental analysis, infrared spectroscopy as well as multinuclear NMR (^1^H, ^13^C, ^14^N, and ^15^N) spectroscopy. The thermal behavior was determined by DTA measurement, and the sensitivities towards external stimuli were determined. Like other azido alkyl‐tetrazoles, it performs well as a ligand in the formation of energetic coordination compounds. Here, the ECCs of copper(II) nitrate (**4**), chlorate (**9**), perchlorate (**7**), azide (**13**), manganese(II) (**5**), iron(II) (**6**), and zinc(II) perchlorate (**8**) were synthesized. The ECCs of copper(II) nitroaromatic salts (**10**–**12**), as well as that of silver(I) fulminate (**14 a/b**) were prepared. Suitable crystals were analyzed by single‐crystal X‐ray diffraction experiments, giving insight into the molecular structure of the ligand itself, as well as its coordination behavior towards 3d and 4d metals. The successful syntheses of the ECCs are supported by elemental analysis as well as infrared spectroscopy. Analysis of the sensitivities towards external stimuli showed the high sensitivity (impact: 2 J, friction: 1 N) of the ligand, which is generally increased by the formation of ECCs. While the impact sensitivity of the ligand was increased by the formation of ECCs (<1 J (**5**–**14**)), the friction sensitivity can be decreased by forming the copper(II) picrate (360 N), styphnate (192 N), and trinitrophloroglycinolate (45 N) complexes. The same trend was observed for the thermal stability of **3** (177 °C). Analyzing the thermal stabilities of the ECCs by DTA, showed decreasing thermal stabilities of the ECCs compared to the ligand itself. Initiation testing of the ligand itself and the ECC of copper(II) trinitrophloroglycinolate (**12**) showed no DDT, thus no initiation of the PETN sample. Testing the perchlorate salts of manganese, copper, and zinc showed a DDT with a positive initiation of PETN.

## Experimental Section

*CAUTION*! This paper contains a large amount of extremely sensitive compounds. Full‐body protection containing Kevlar® gloves, Kevlar® sleeves, face shield, leather coat and ear protection must be worn when manipulating those compounds! The equipment used must be earthed!

**1‐(2‐Hydroxymethyl)‐5*H*‐tetrazole** ^[30]^ **(1)**: 1,5*H*‐tetrazole (20 g, 285 mmol), synthesized according to literature,[^35^, ^36^] and sodium hydroxide (0.5 g, 12.5 mmol) were dissolved in water (200 mL), and formalin solution (37 %, 66 mL, 917 mmol) was added. The reaction mixture was stirred over night at room temperature and was extracted into ethyl acetate (3x400 mL). The organic phase was dried over MgSO_4_, and the solvent was removed in vacuo, resulting in crude isomeric mixture **1** as a colorless oil in good yield (22.1 g, 221 mmol, 80 %). ^1^H NMR ([D_6_]DMSO, 25 °C): **1 a** *δ*=9.45 (s, C*H*), 5.77 (s, C*H*_2_); **1 b** *δ*=8.97 (s, C*H*), 5.92 (s, C*H*_2_).

**(Tetrazol‐1‐yl)methyl methanesulfonate (2 a)**: Crude isomeric mixture **1** (5.0 g, 50 mmol) was suspended in CH_2_Cl_2_ (100 mL), cooled to 0 °C. Methanesulfonyl chloride (6.87 g, 60 mmol, 1.2 equiv.) was added, the reaction mixture was stirred for 1 h while cooling, and pyridine (5.93 g, 75 mmol, 1.5 equiv.) was added, keeping the temperature below 5 °C. The reaction mixture was allowed to slowly warm and was stirred for 18 h at room temperature. The reaction mixture was then transferred to a separatory funnel and washed with water (100 mL), hydrochloric acid (2 m, 50 mL), and water (50 mL). Colorless crystals of compound **2 a** slowly start to crystallize from the aqueous phase in moderate yield (3.12 g, 17.5 mmol, 35 %). The organic phase was dried over MgSO_4_, and the solvent was removed in vacuo, leaving a colorless oil consisting of compounds **2 a** and **2 b**, which were separated by flash column chromatography over silica with *n*‐hexane/ethyl acetate (50 : 50) as the eluting solvent. After removing the solvent in vacuo, additional compound **2 a** was obtained in a yield of 13 %, whereas crude compound **2 b** was still obtained. ^1^H NMR ([D_6_]acetone, 25 °C): **2 a** *δ*=9.43 (s, C*H*), 6.66 (s, C*H*_2_), 3.27 ppm (s, C*H*_3_); **2 b** *δ*=8.95 (s, C*H*), 6.77 (s, C*H*_2_), 3.24 ppm (s, C*H*_3_). ^13^C NMR ([D_6_]acetone, 25 °C): **2 a** *δ*=145.5 (s, C*H*), 72.7 (s, C*H*_2_), 39.0 ppm (s, C*H*_3_).

**1‐(Azidomethyl)‐5*H*‐tetrazole (AzMT, 3)**: Compound **2 a** (350 mg, 1.96 mmol) and sodium azide (383 g, 5.89 mmol) were dissolved in a mixture of water (10 mL) and acetone (10 mL), and the mixture was refluxed for 18 h at 75 °C. After cooling to room temperature, water (40 mL) was added, and the mixture was extracted into CH_2_Cl_2_ (3×50 mL). The organic phase was dried over MgSO_4_, the solvent was removed in vacuo, and 1‐(azidomethyl)‐5*H*‐tetrazole (**3**) was obtained as an off‐white solid in quantitative yield (243 mg, 1.94 mmol, 99 %). ^1^H NMR ([D_6_]DMSO, 25 °C): *δ*=9.60 (s, C*H*), 6.02 ppm (s, C*H*_2_); ^13^C NMR ([D_6_]DMSO, 25 °C): *δ*=144.2 (s, *C*H), 60.5 ppm (s, *C*H_2_); ^14^N NMR ([D_6_]DMSO, 25 °C): *δ*=−135.6.2 (br s, N_β_), −160.4 ppm (br s, N_γ_); ^15^N NMR ([D_6_]DMSO, 25 °C): *δ*=14.4 (d, N_(3)_), −14.0 (s, N_(2)_), −50.4 (d, N_(4)_), −136.3 (t, N_(α)_), −141.9 (d, N_(1)_), −158.9 (s, N_(γ)_), −302.1 ppm (s, N_(β)_); elemental analysis calcd. (%) for C_2_H_3_N_7_ (125.10): C 19.20, H 2.42, N 78.38; found C 19.02, H 2.32, N 76.00; DTA (5 °C min^−1^): 54 °C (*T*_endo_), 177 °C (*T*_exo_); BAM drop hammer: 2 J; BAM friction tester: 1 N; ESD: 540 mJ; IR (ATR, cm^−1^): 3154 (m), 3048 (w), 2979 (w), 2460 (w), 2236 (w), 2153 (s), 2106 (vs), 1819 (vw), 1762 (w), 1478 (s), 1443 (m), 1428 (m), 1370 (w), 1317 (w), 1294 (s), 1263 (m), 1230 (vs), 1161 (vs), 1100 (vs), 1026 (w), 1000 (w), 951 (m), 911 (s), 881 (s), 754 (s), 716 (s), 655 (vs), 562 (s), 416 (w).

### Synthesis of energetic coordination compounds

*General procedure*: Compound **3** (125 mg, 1 mmol) was dissolved in water at 60–80 °C. In a second container, the corresponding equivalents of metal salt (**4**–**9**: 0.16 mmol, **10**–**12**: 0.5 mmol, **13** and **14**: 1 mmol) was dissolved in water (**4**–**13**) or acetonitrile (**14**) at 60–80 °C. After all solids dissolved, the solution containing compound **3** was added to the metal salt solution with stirring. After stirring for 5 min at 60 °C, any undissolved solids were filtered off, and the solution was left to crystallize for compounds **4**–**12**, and **14 a/b**. After the solvent was evaporated, the remaining solids were dried under high vacuum for 1 h. Compound **13** was obtained by combining the two solutions, stirring for 5 min at 60 °C, filtering off the precipitate and washing with cold water.

**[Cu(AzMT)_6_](NO_3_)_2_ (4)**: Obtained as blue platelets in good yield (124.6 mg, 0.133 mmol, 83 %). Elemental analysis calcd. (%) for C_12_H_18_CuN_44_O_6_ (938.12): C 15.36, H 1.93, N 65.70; found C 15.46, H 1.94, N 62.97; DTA (5 °C min^−1^): 60 °C (*T*_endo_), 123 °C (*T*_exo_); BAM drop hammer: 2 J; BAM friction tester: 15 N; ESD: >1500 mJ. IR (ATR, cm^−1^): 3086 (m), 3043 (m), 2984 (m), 2454 (w), 2394 (w), 2352 (w), 2315 (w), 2156 (s), 2106 (s), 1588 (m), 1501 (m), 1447 (m), 1342 (vs), 1290 (s), 1277 (s), 1230 (s), 1169 (s), 1162 (s), 1100 (vs), 1043 (m), 997 (m), 913 (s), 829 (m), 781 (m), 748 (s), 716 (m), 648 (s), 561 (m), 421 (w), 413 (w).

**[Mn(AzMT)_6_](ClO_4_)_2_ (5)**: Obtained as white platelets in quantitative yield (160.5 mg, 0.16 mmol, 100 %). Elemental analysis calcd. (%) for C_12_H_18_Cl_2_MnN_42_O_8_ (1004.40): C 14.35, H 1.81, N 58.87; found C 14.79, H 1.91, N 58.01; DTA (5 °C min^−1^): 137 °C (*T*_exo_); BAM drop hammer: <1 J; ball drop impact: 25 mJ; BAM friction tester: 0.5 N; ESD: 740 mJ; IR (ATR, cm^−1^): 3128 (m), 3096 (m), 3038 (m), 2982 (m), 2157 (s), 2105 (s), 1586 (m), 1497 (s), 1448 (m), 1373 (m), 1311 (m), 1283 (m), 1232 (s), 1174 (s), 1100 (vs), 1041 (m), 978 (s), 935 (s), 913 (s), 747 (s), 713 (m), 647 (s), 622 (s), 604 (m), 561 (m), 479 (m), 425 (w), 420 (w).

**[Fe(AzMT)_6_](ClO_4_)_2_ (6)**: Obtained as an orange‐brown solid in very good yield (149.6 mg, 0.149 mmol, 99 %). Elemental analysis calcd. (%) for C_12_H_18_Cl_2_FeN_42_O_8_ (1005.31): C 14.34, H 1.80, N 58.52; found C 15.10, H 2.02, N 58.86; DTA (5 °C min^−1^): 127 °C (*T*_exo_); BAM drop hammer: <1 J; ball drop impact: 16 mJ; BAM friction tester: <0.1 N; ESD: 380 mJ; IR (ATR, cm^−1^): 3135 (w), 3043 (vw), 2986 (vw), 2163 (m), 2106 (s), 1497 (m), 1440 (w), 1375 (w), 1314 (m), 1285 (m), 1237 (s), 1172 (m), 1075 (vs), 1039 (m), 983 (m), 914 (s), 896 (m), 746 (m), 712 (m), 655 (s), 651 (s), 622 (s), 560 (m), 425 (vw), 413 (w), 407 (vw).

**[Cu(AzMT)_6_](ClO_4_)_2_ (7)**: Obtained as blue platelets in quantitative yield (162 mg, 0.16 mmol, 100 %). Elemental analysis calcd. (%) for C_12_H_18_Cl_2_CuN_42_O_8_ (1013.01): C 14.23, H 1.79, N 58.07; found C 14.33, H 1.97, N 57.50; DTA (5 °C min^−1^): 133 °C (*T*_exo_); BAM drop hammer:<1 J; ball drop impact: 25 mJ; BAM friction tester: <0.1 N; ESD: 480 mJ; IR (ATR, cm^−1^): 3136 (m), 3045 (w), 2992 (w), 2155 (m), 2106 (s), 1591 (vw), 1556 (vw), 1503 (m), 1447 (w), 1375 (w), 1284 (m), 1236 (m), 1175 (m), 1100 (s), 1074 (vs), 992 (m), 909 (m), 747 (m), 711 (m), 655 (s), 647 (s), 622 (vs), 560(m).

**[Zn(AzMT)_6_](ClO_4_)_2_ (8)**: Obtained as white solid in good yield (141.3 mg, 0.139 mmol, 87 %). Elemental analysis calcd. (%) for C_12_H_18_Cl_2_N_42_O_8_Zn (1014.84): C 14.20, H 1.79, N 57.97; found C 14.60, H 1.86, N 57.37; DTA (5 °C min^−1^): 130 °C (*T*_exo_); BAM drop hammer: <1 J; ball drop impact: 20 mJ; BAM friction tester: <0.1 N; ESD: 380 mJ; IR (ATR, cm^−1^): 3136 (w), 3048 (vw), 2986 (vw), 2163 (m), 2108 (s), 1793 (vw), 1498 (m), 1442 (w), 1375 (w), 1315 (w), 1284 (m), 1238 (m), 1175 (m), 1074 (vs), 1040 (m), 985 (m), 915 (m), 897 (m), 746 (m), 711 (m), 656 (m), 647 (m), 622 (s), 560 (m), 416 (w).

**[Cu(AzMT)_6_](ClO_3_)_2_ (9)**: Obtained as light‐blue blocks in good yield (146 mg, 0.149 mmol, 93 %). Elemental analysis calcd. (%) for C_12_H_18_Cl_2_CuN_42_O_6_ (981.01): C 14.69, H 1.85, N 59.97; found C 14.94, H 2.04, N 58.84; DTA (5 °C min^−1^): 112 °C (*T*_endo_), 123 °C (*T*_exo_); BAM drop hammer: <1 J; BAM friction tester: <0.1 N; ESD: 380 mJ; IR (ATR, cm^−1^): 3098 (s), 3039 (m), 2985 (m), 2458 (w), 2227 (w), 2155 (s), 2104 (s), 1588 (w), 1499 (m), 1448 (m), 1373 (m), 1310 (m), 1292 (m), 1281 (m), 1230 (s), 1176 (s), 1163 (s), 1100 (vs), 1042 (m), 958 (vs), 935 (s), 912 (vs), 775 (m), 747 (m), 715 (m), 646 (s), 603 (s), 562 (m), 479 (s), 426 (w), 411 (w).

**[Cu(PA)_2_(AzMT)_2_] (10)**: Obtained as green platelets in good yield (338.8 mg, 0.44 mmol, 88 %). Elemental analysis calcd. (%) for C_16_H_10_CuN_20_O_14_ (769.93): C 24.96, H 1.31, N 36.39; found C 24.84, H 1.29, N 35.97; DTA (5 °C min^−1^): 165 °C (*T*_exo_); BAM drop hammer: <1 J; BAM friction tester: >360 N; ESD: >1500 mJ. IR (ATR, cm^−1^): 3147 (w), 3094 (w), 3060 (w), 2158 (m), 2106 (s), 1608 (s), 1574 (s), 1542 (s), 1517 (s), 1493 (s), 1420 (m), 1349 (s), 1321 (s), 1276 (s), 1239 (s), 1163 (vs), 1092 (s), 1037 (m), 1012 (m), 988 (m), 941 (m), 933 (m), 910 (s), 899 (s), 847 (m), 825 (m), 788 (s), 741 (s), 707 (vs), 659 (s), 542 (s), 515 (m), 460 (m), 427 (m), 418 (m).

**[Cu(HTNR)_2_(AzMT)_2_] (11)**: Obtained as dark green blocks in moderate yield (312.8 mg, 0.39 mmol, 78 %). Elemental analysis calcd. (%) for C_16_H_10_CuN_20_O_16_ (801.93): C 23.96, N 1.26, N 34.93,; found C 24.01, H 1.60, N 34.78; DTA (5 °C min^−1^): 154 °C (*T*_exo_); BAM drop hammer: <1 J; BAM friction tester: 192 N; ESD: >1500 mJ. IR (ATR, cm^−1^): 3135 (w), 3052 (w), 2149 (m), 2098 (m), 1627 (m), 1561 (s), 1520 (s), 1490 (m), 1477 (m), 1456 (s), 1443 (m), 1368 (s), 1334 (s), 1321 (m), 1307 (s), 1274 (vs), 1247 (s), 1222 (s), 1204 (s), 1173 (s), 1160 (s), 1094 (vs), 1037 (m), 987 (s), 948 (m), 931 (m), 915 (s), 891 (s), 829 (w), 783 (m), 775 (m), 763 (m), 747 (m), 734 (s), 705 (s), 697 (vs), 646 (vs), 578 (m), 562 (m), 527 (m), 464 (m), 456 (m), 421 (m).

**[Cu(HTNPG)(AzMT)_2_] (12)**: Obtained as dark brown blocks in moderate yield (203.4 mg, 0.36 mmol, 71 %). Elemental analysis calcd. (%) for C_10_H_7_CuN_17_O_9_ (572.82): C 20.97, H 1.23, N 41.57; found C 21.00, H 1.66, N 39.33; DTA (5 °C min^−1^): 151 °C (*T*_exo_); BAM drop hammer: <1 J; BAM friction tester: 45 N; ESD: 1220 mJ. IR (ATR, cm^−1^): 3142 (m), 3043 (w), 2984 (w), 2163 (m), 2112 (s), 2105 (s), 1645 (m), 1589 (m), 1569 (s), 1521 (s), 1515 (s), 1503 (s), 1494 (s), 1447 (m), 1436 (m), 1404 (m), 1375 (m), 1337 (s), 1318 (s), 1288 (s), 1237 (s), 1164 (vs), 1092 (vs), 1032 (m), 1008 (m), 1001 (m), 984 (m), 916 (s), 905 (m), 872 (w), 833 (m), 820( m), 805 (m), 786 (m), 760 (m), 740 (m), 710 (s), 694 (s), 645 (s), 559 (m), 503 (m), 493 (m), 457 (w), 418 (w).

**[Cu(N_3_)_2_(AzMT)] (13)**: Obtained as brown powder in moderate yield (171.8 mg, 0.63 mmol, 63 %). Elemental analysis calcd. (%) for C_2_H_3_CuN_13_ (272.68): C 8.81, H 1.11, N 66.78; found C 8.92, H 1.46, N 63.60; DTA (5 °C min^−1^): 147 °C (*T*_exo_); BAM drop hammer: <1 J; BAM friction tester: <0.1 N; ESD: 38 mJ. IR (ATR, cm^−1^): 3104 (m), 3047 (w), 2463 (w), 2158 (m), 2080 (vs), 2047 (vs), 1501 (s), 1447 (m), 1377 (m), 1340 (m), 1309 (m), 1286 (s), 1239 (s), 1171 (vs), 1101 (s), 1039 (m), 1024 (m), 993 (m), 909 (s), 895 (s), 759 (s), 713 (m), 689 (m), 654 (vs), 597 (m), 587 (s), 562 (m), 403 (s).

**[Ag_4_(CNO)_4_(AzMT)_2_] (14 a)**: Obtained as colorless platelets in moderate yield (280.4 mg, 0.66 mmol, 66 %). Elemental analysis calcd. (%) for C_4_H_3_Ag_2_N_9_O_2_ (424.87): C 11.31, H 0.71, N 29.67; found C 11.37, H 0.86, N 29.90; DTA (5 °C min^−1^): 109 °C (*T*_exo_); BAM drop hammer: <1 J; BAM friction tester: <0.1 N; ESD: 10 mJ; IR (ATR, cm^−1^): 3220 (w), 3134 (m), 3032 (w), 2960 (w), 2458 (w), 2303 (w), 2292 (w), 2246 (w), 2154 (m), 2104 (vs), 2090 (vs), 1818 (w), 1487 (m), 1449 (m), 1439 (w), 1376 (w), 1301 (m), 1276 (m), 1235 (s), 1152 (vs), 1130 (vs), 1099 (s), 1035 (m), 1008 (m), 976 (m), 909 (s), 885 (s), 767 (w), 746 (m), 710 (m), 654 (s), 563 (m), 498 (w), 481 (m), 462 (m), 418 (w), 405 (w).

## Conflict of interest

The authors declare no conflict of interest.

1

## Supporting information

As a service to our authors and readers, this journal provides supporting information supplied by the authors. Such materials are peer reviewed and may be re‐organized for online delivery, but are not copy‐edited or typeset. Technical support issues arising from supporting information (other than missing files) should be addressed to the authors.

Supporting Information

## Data Availability

The data that support the findings of this study are available in the supplementary material of this article.

## References

[1] J. Wilbrand , Justus Liebigs Ann. Chem. 1863, 128, 178–179.

[2] A. Cahours, A. Sobrero *Justus Liebigs Ann. Chem*. **1848**, *64*, 396–398.

[3] D. Kumar , Y. Tang , C. He , G. H. Imler , D. A. Parrish , J. n. M. Shreeve , Chem. Eur. J. 2018, 24, 17220–17224. 30231192 10.1002/chem.201804418

[4] E. C. Johnson , T. A. Reid , C. W. Miller , J. J. Sabatini , R. C. Sausa , E. F. C. Byrd , J. A. Orlicki , ChemPlusChem 2021, 86, 875–878. 34114374 10.1002/cplu.202100175

[5] E. C. Johnson , J. J. Sabatini , D. E. Chavez , L. A. Wells , J. E. Banning , R. C. Sausa , E. F. C. Byrd , J. A. Orlicki , ChemPlusChem 2020, 85, 237–239. 31961517 10.1002/cplu.201900710

[6] J. Fronabarger, A. Schuman, R. Chapman, W. Fleming, W. Sanborn, T. Massis, in *31st Joint Propulsion Conference and Exhibit*, AIAA, July **1995**..

[7] T. W. Myers , K. E. Brown , D. E. Chavez , R. J. Scharff , J. M. Veauthier , Inorg. Chem. 2017, 56, 2297–2303. 28145693 10.1021/acs.inorgchem.6b02998

[8] M. L. Gettings , M. T. Thoenen , E. F. C. Byrd , J. J. Sabatini , M. Zeller , D. G. Piercey , Chem. Eur. J. 2020, 26, 14530–14535. 32567079 10.1002/chem.202002664

[9] S. Manzoor , Q.-u.-n. Tariq , X. Yin , J.-G. Zhang , Def. Tech. 2021, 17, 1995–2010.

[10] A. A. Larin , L. L. Fershtat , Energ. Mater. Front. 2021, 2, 3–13.

[11] A. A. Larin , N. V. Muravyev , A. N. Pivkina , K. Y. Suponitsky , I. V. Ananyev , D. V. Khakimov , L. L. Fershtat , N. N. Makhova , Chem. Eur. J. 2019, 25, 4225–4233. 30644611 10.1002/chem.201806378

[12] Q. Wu , Q. Li , K. Li , H. Li , B. Kou , Z. Hang , W. Zhu , Can. J. Chem. 2018, 96, 459–465.

[13] S. R. Yocca , M. Zeller , E. F. C. Byrd , D. G. Piercey , J. Mater. Chem. A 2022, 10, 1876–1884.

[14] P. He , J.-G. Zhang , X. Yin , J.-T. Wu , L. Wu , Z.-N. Zhou , T.-L. Zhang , Chem. Eur. J. 2016, 22, 7670–7685. 27061423 10.1002/chem.201600257

[15] K. Y. Titenkova , A. V. Shaferov , A. A. Larin , M. A. Epishina , A. S. Kulikov , I. V. Ananyev , L. L. Fershtat , Tetrahedron 2022, 103, 132563.

[16] T. Liu , Y.-G. Ji , L. Wu , Org. Biomol. Chem. 2019, 17, 2619–2623. 30766975 10.1039/c9ob00169g

[17] A. Qian , Y. Zheng , R. Wang , J. Wei , Y. Cui , X. Cao , Y. Yang , Bioorg. Med. Chem. Lett. 2018, 28, 344–350. 29289430 10.1016/j.bmcl.2017.12.040

[18] T. M. Klapötke , M. Kofen , L. Schmidt , J. Stierstorfer , M. H. H. Wurzenberger , Chem. Asian J. 2021, 16, 3001–3012. 34411440 10.1002/asia.202100714 PMC8518496

[19] N. Szimhardt , M. H. H. Wurzenberger , A. Beringer , L. J. Daumann , J. Stierstorfer , J. Mater. Chem. A 2017, 5, 23753–23765.

[20] N. Szimhardt , M. H. H. Wurzenberger , L. Zeisel , M. S. Gruhne , M. Lommel , J. Stierstorfer , J. Mater. Chem. A 2018, 6, 16257–16272.

[21] M. H. H. Wurzenberger , B. R. G. Bissinger , M. Lommel , M. S. Gruhne , N. Szimhardt , J. Stierstorfer , New J. Chem. 2019, 43, 18193–18202.

[22] M. H. H. Wurzenberger , S. M. J. Endraß , M. Lommel , T. M. Klapötke , J. Stierstorfer , ACS Appl. Energ. Mater. 2020, 3, 3798–3806.

[23] M. H. H. Wurzenberger , M. S. Gruhne , M. Lommel , N. Szimhardt , T. M. Klapötke , J. Stierstorfer , Chem. Asian J. 2019, 14, 2018–2028. 30942533 10.1002/asia.201900269

[24] G. Bélanger-Chabot , S. M. Kaplan , P. Deokar , N. Szimhardt , R. Haiges , K. O. Christe , Chem. Eur. J. 2017, 23, 13087–13099. 28590071 10.1002/chem.201701690

[25] R. Haiges , K. O. Christe , Dalton Trans. 2015, 44, 10166–10176. 25826267 10.1039/c5dt00291e

[26] A. R. Katritzky , C. N. Fali , I. V. Shcherbakova , S. V. Verin , J. Heterocycl. Chem. 1996, 33, 335–339.

[27] O. N. Verkhozina , V. N. Kizhnyaev , L. I. Vereshchagin , A. V. Rokhin , A. I. Smirnov , Russ. J. Org. Chem. 2003, 39, 1792–1796.

[28] D. A. Chaplygin , A. A. Larin , N. V. Muravyev , D. B. Meerov , E. K. Kosareva , V. G. Kiselev , A. N. Pivkina , I. V. Ananyev , L. L. Fershtat , Dalton Trans. 2021, 50, 13778–13785. 34505609 10.1039/d1dt02688g

[29] L. Y. Peng Lei , Y. Yuzhang , L. Wei , Z. Xuejiao , P. Siping , Chin. J. Org. Chem. 2012, 32, 667–676.

[30] I. V. Tselinskii , A. A. Mel′nikov , L. G. Varyagina , I. G. Zhigadlova , Chem. Heterocycl. Compd. 1983, 19, 341–343.

[31] M. Sućeska, *EXPLO5 Version 6.05 User's Guide*, Zagreb, **2018**.

[32] M. H. H. Wurzenberger , M. Lommel , M. S. Gruhne , N. Szimhardt , J. Stierstorfer , Angew. Chem. Int. Ed. 2020, 59, 12367–12370; 10.1002/anie.202002823 PMC7383744 32237192

[33] M. H. H. Wurzenberger , M. S. Gruhne , M. Lommel , V. Braun , N. Szimhardt , J. Stierstorfer , Inorg. Chem. 2020, 59, 17875–17879. 33270434 10.1021/acs.inorgchem.0c03027

[34] H. Schmidbaur , A. Schier , Angew. Chem. Int. Ed. 2015, 54, 746–784; 10.1002/anie.201405936 25393553

[35] R. Bronisz , Inorg. Chim. Acta 2002, 215–220.

[36] T. M. Klapötke , M. Stein , J. Stierstorfer , Z. Anorg. Allg. Chem. 2008, 634, 1711–1723.

