# Supplementary material for: 1‐(Azidomethyl)‐5H‐Tetrazole: A Powerful New Ligand for Highly Energetic Coordination Compounds
Source: Chemistry. 2022 May 25;28(38):e202200492. doi: 10.1002/chem.202200492 (PMC9400890; doi:10.1002/chem.202200492)
Supplement: Supplementary file 1 — Supporting Information [file CHEM-28-0-s001.pdf]

# Chemistry–A European Journal

Supporting Information


## 1-(Azidomethyl)-5*H*-Tetrazole: A Powerful New Ligand for Highly Energetic Coordination Compounds


Moritz Kofen, Marcus Lommel, Maximilian H. H. Wurzenberger, Thomas M. Klapötke, and Jörg Stierstorfer*

**Table of Contents**

1. Compounds Overview
2. Single Crystal X-Ray Diffraction
3. Computations
4. NMR Spectroscopy of **1–3**
5. IR Spectroscopy of **1–14a/b**
6. Hot Plate and Hot Needle Testing
7. General Methods
8. References

## 1. Overview of compounds

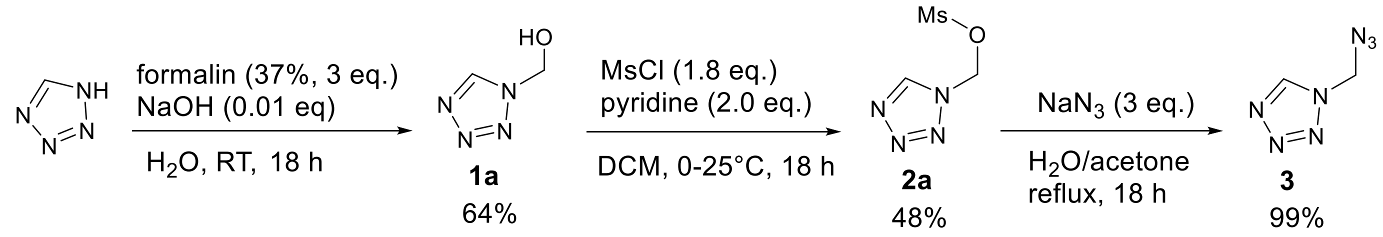


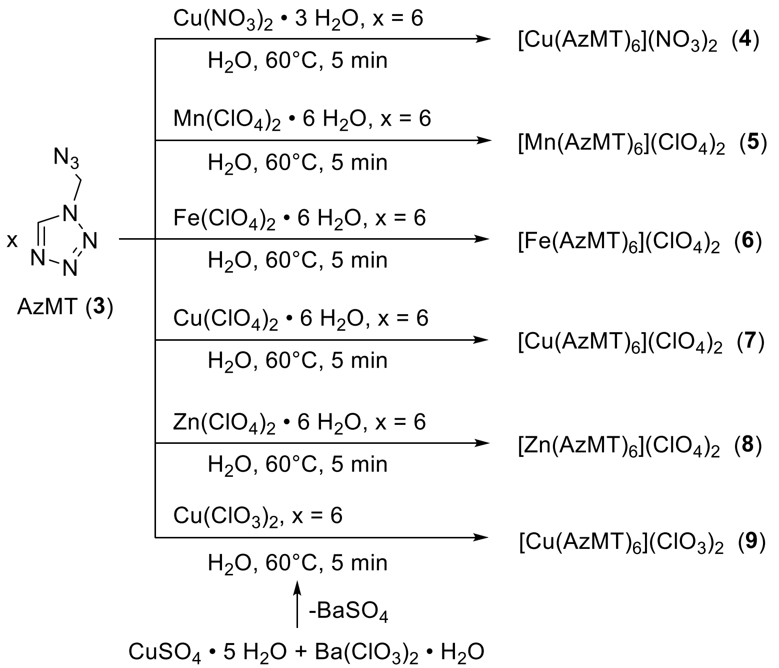


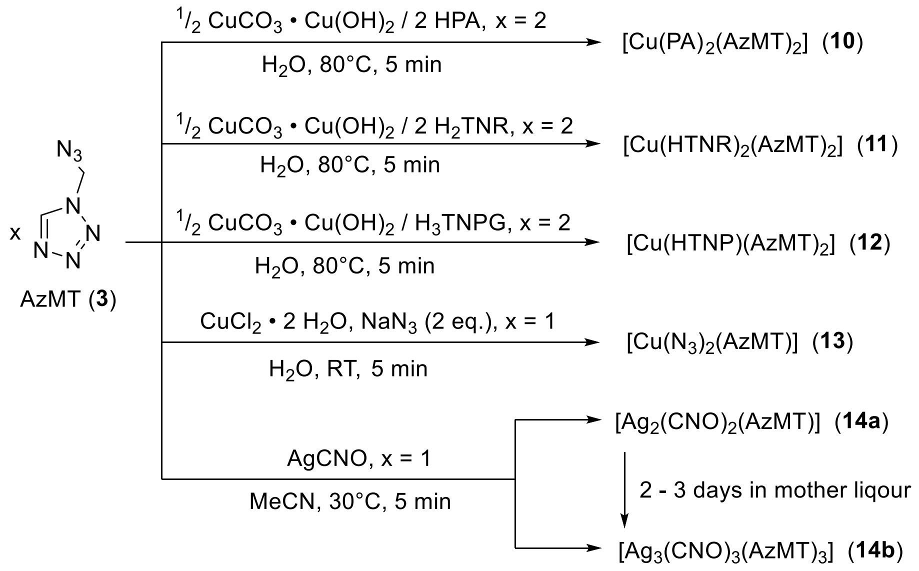

## 2. Single Crystal X-Ray Diffraction

For all crystalline compounds an Oxford Xcalibur3 diffractometer with a CCD area detector or Bruker D8 Venture TXS diffractometer equipped with a multilayer monochromator, a Photon 2 detector, and a rotating-anode generator were employed for data collection using Mo-$K_\alpha$ radiation ($\lambda$ = 0.7107 Å). On the Oxford device, data collection and reduction were carried out using the CrysAlisPRO software.[S1] On the Bruker diffractometer, the data were collected with the Bruker Instrument Service v3.0.21, the data reduction was performed using the SAINT V8.18C software (Bruker AXS Inc., 2011). The structures were solved by direct methods (SIR-92,[S2] SIR-97,[S3,S4] SHELXS-97[S5,S6] or SHELXT[S7]), refined by full-matrix least-squares on *F*2 (SHELXL[S5,S6]) and finally checked using the PLATON software[S8] integrated in the WinGX[S7,S9] or Olex2[S8] software suite. The non-hydrogen atoms were refined anisotropically and the hydrogen atoms were located and freely refined. The absorptions were corrected by a SCALE3 ABSPACK or SADABS Bruker APEX3 multi-scan method.[S11,S12] All DIAMOND2 plots are shown with thermal ellipsoids at the 50% probability level and hydrogen atoms are shown as small spheres of arbitrary radius.

**Table S1**. Crystallographic data and structure refinement details for compounds **3**, **4**, and **9.**

| | **AzMT (3)** | **4** | **9** |
|---|---|---|---|
| Formula | $C_2H_3N_7$ | $C_{12}H_{18}CuN_{44}O_6$ | $C_{12}H_{18}CuN_{42}Cl_2O_6$ |
| FW [g mol$^{-1}$] | 125.11 | 938.24 | 981.12 |
| Crystal system | orthorhombic | monoclinic | triclinic |
| Space group | *Pbca* (No. 61) | *Cc* (No. 9) | *P*-1 (No. 2) |
| Color / Habit | colorless block | blue platelet | blue block |
| Size [mm] | 0.07 x 0.10 x 0.36 | 0.07 x 0.17 x 0.30 | 0.27 x 0.42 x 0.43 |
| a [Å] | 6.7828(7) | 10.0179(7) | 10.1101(6) |
| b [Å] | 11.3306(19) | 17.9322(9) | 10.2068(5) |
| c [Å] | 13.6136(17) | 20.4520(14) | 20.841(1) |
| α [°] | 90 | 90 | 86.942(4) |
| β [°] | 90 | 95.777(6) | 80.881(5) |
| γ [°] | 90 | 90 | 60.690(6) |
| *V* [Å$^3$] | 1046.3(2) | 3655.4(4) | 1850.9(2) |
| Z | 8 | 4 | 2 |
| $\rho_{calc.}$ [g cm$^{-3}$] | 1.589 | 1.705 | 1.760 |
| μ [mm$^{-1}$] | 0.124 | 0.699 | 0.832 |
| F(000) | 512 | 1900 | 990 |
| $\lambda_{MoK\alpha}$ [Å] | 0.71073 | 0.71073 | 0.71073 |
| T [K] | 110 | 103 | 114 |
| θ Min-Max [°] | 3.0, 26.0 | 2.0, 26.4 | 2.3, 26.4 |
| Dataset | -7: 8; -9: 13; -16: 16 | -12: 12; -22: 22; -25: 25 | -12: 12; -7: 12; -22: 26 |
| Reflections collected | 6247 | 24281 | 10869 |
| Independent refl. | 1031 | 7447 | 7485 |
| $R_{int}$ | 0.077 | 0.082 | 0.024 |
| Observed reflections | 682 | 5490 | 6207 |
| Parameters | 94 | 569 | 571 |
| $R_1$ (obs)[a] | 0.0488 | 0.0554 | 0.0404 |
| w$R_2$ (all data)[b] | 0.0984 | 0.0879 | 0.1331 |
| *S* [c] | 1.07 | 1.02 | 0.92 |
| Resd. dens [e Å$^{-3}$] | -0.19, 0.21 | -0.49, 0.96 | -0.45, 0.62 |
| Device type | Oxford Xcalibur3 | Oxford Xcalibur3 | Oxford Xcalibur3 |
| Solution | SHELXT | SHELXT | SHELXT |
| Refinement | SHELXL-2018 | SHELXL-2018 | SHELXL-2018 |
| Absorption correction | multi-scan | multi-scan | multi-scan |

[a]$R_1 = \Sigma||F_0|-|F_c||/\Sigma|F_0|$; [b]$wR_2 = [\Sigma[w(F_0^2-F_c^2)^2]/\Sigma[w(F_0)^2]]^{1/2}$; $w = [\sigma c^2(F_0^2)+(xP)^2+yP]^{-1}$ and $P=(F_0^2+2F_c^2)/3$; [c]$S = \{\Sigma[w(F_o^2-F_c^2)^2]/(n-p)\}^{1/2}$ (n = number of reflections; p = total number of parameters).

**Table S2**. Crystallographic data and structure refinement details for compounds **10**, **13**, and **14a**

| | **10** | **13** | **14a** |
|---|---|---|---|
| Formula | $C_{16}H_{10}CuN_{20}O_{14}$ | $C_2H_3CuN_{13}$ | $C_4H_3Ag_2N_9O_2$ |
| FW [g mol$^{-1}$] | 769.98 | 272.71 | 424.89 |
| Crystal system | monoclinic | monoclinic | monoclinic |
| Space group | $P2_1/c$ (No. 14) | $P2_1/c$ (No. 14) | $I2/a$ (No. 15) |
| Color / Habit | green block | brown plate | colorless platelet |
| Size [mm] | 0.24 x 0.38 x 0.66 | 0.02 x 0.24 x 0.63 | 0.02 x 0.10 x 0.40 |
| a [Å] | 9.7058(11) | 12.1752(9) | 12.8907(7) |
| b [Å] | 18.0227(17) | 5.7388(4) | 5.5319(2) |
| c [Å] | 7.7834(8) | 13.0612(9) | 29.175(2) |
| α [°] | 90 | 90 | 90 |
| β [°] | 90.975(10) | 93.975(7) | 91.266(5) |
| γ [°] | 90 | 90 | 90 |
| *V* [Å$^3$] | 1361.3(2) | 910.40(11) | 2080.0(2) |
| Z | 2 | 4 | 8 |
| $\rho_{calc.}$ [g cm$^{-3}$] | 1.878 | 1.990 | 2.714 |
| μ [mm$^{-1}$] | 0.913 | 2.399 | 3.773 |
| F(000) | 774 | 540 | 1600 |
| $\lambda_{MoK\alpha}$ [Å] | 0.71073 | 0.71073 | 0.71073 |
| T [K] | 103 | 102 | 105 |
| θ Min-Max [°] | 2.3, 26.4 | 3.1, 29.1 | 2.8, 26.4 |
| Dataset | -12: 12 ; -22: 22 ; -7: 9 | -16: 15 ; -7: 4 ; -16: 17 | -16: 13 ; -6: 6 ; -36: 34 |
| Reflections collected | 11796 | 4057 | 6587 |
| Independent refl. | 2786 | 2079 | 2118 |
| $R_{int}$ | 0.046 | 0.029 | 0.036 |
| Observed reflections | 2130 | 1681 | 1632 |
| Parameters | 261 | 145 | 154 |
| $R_1$ (obs)[a] | 0.0388 | 0.0364 | 0.0368 |
| w$R_2$ (all data)[b] | 0.1007 | 0.0903 | 0.0694 |
| *S* [c] | 1.02 | 1.04 | 1.06 |
| Resd. dens [e Å$^{-3}$] | -0.26, 0.43 | -0.44, 0.65 | -0.55, 0.64 |
| Device type | Oxford Xcalibur3 | Oxford Xcalibur3 | Oxford Xcalibur3 |
| Solution | SHELXT | SHELXT | SHELXT |
| Refinement | SHELXL-2018 | SHELXL-2018 | SHELXL-2018 |
| Absorption correction | multi-scan | multi-scan | multi-scan |

[a]$R_1 = \Sigma||F_0|-|F_c||/\Sigma|F_0|$; [b]$wR_2 = [\Sigma[w(F_0^2-F_c^2)^2]/\Sigma[w(F_0)^2]]^{1/2}$; $w = [\sigma c^2(F_0^2)+(xP)^2+yP]^{-1}$ and $P=(F_0^2+2F_c^2)/3$; [c]$S = \{\Sigma[w(F_o^2-F_c^2)^2]/(n-p)\}^{1/2}$ (n = number of reflections; p = total number of parameters).

**Table S3**. Crystallographic data and structure refinement details for compound **14b**.

| | **14b** |
|---|---|
| Formula | $C_9H_9Ag_3N_{24}O_3$ |
| FW [g mol$^{-1}$] | 825.01 |
| Crystal system | triclinic |
| Space group | *P*-1 (No. 2) |
| Color / Habit | colorless rod |
| Size [mm] | 0.02 x 0.03 x 0.12 |
| a [Å] | 3.8316(7) |
| b [Å] | 18.221(3) |
| c [Å] | 19.080(3) |
| α [°] | 117.720(4) |
| β [°] | 94.189(5) |
| γ [°] | 93.814(6) |
| *V* [Å$^3$] | 1168.4(3) |
| Z | 2 |
| $\rho_{calc.}$ [g cm$^{-3}$] | 2.345 |
| μ [mm$^{-1}$] | 2.560 |
| F(000) | 792 |
| $\lambda_{MoK\alpha}$ [Å] | 0.71073 |
| T [K] | 173 |
| θ Min-Max [°] | 3.2, 26.4 |
| Dataset | -4: 4; -22: 22; -23: 23 |
| Reflections collected | 19270 |
| Independent refl. | 4760 |
| $R_{int}$ | 0.038 |
| Observed reflections | 4384 |
| Parameters | 352 |
| $R_1$ (obs)[a] | 0.0214 |
| w$R_2$ (all data)[b] | 0.0464 |
| *S* [c] | 1.07 |
| Resd. dens [e Å$^{-3}$] | -0.34, 0.56 |
| Device type | Bruker D8 Venture TXS |
| Solution | SHELXT |
| Refinement | SHELXL-2018 |
| Absorption correction | multi-scan |

[a]$R_1 = \Sigma||F_0|-|F_c||/\Sigma|F_0|$; [b]$wR_2 = [\Sigma[w(F_0^2-F_c^2)^2]/\Sigma[w(F_0)^2]]^{1/2}$; $w = [\sigma c^2(F_0^2)+(xP)^2+yP]^{-1}$ and $P=(F_0^2+2F_c^2)/3$; [c]$S = \{\Sigma[w(F_o^2-F_c^2)^2]/(n-p)\}^{1/2}$ (n = number of reflections; p = total number of parameters).

## 3. Computations

### 3.1 Computations

All calculations were carried out using the Gaussian G09 program package.[S13] The enthalpies (H) and free energies (G) were calculated using the complete basis set (CBS) method of Petersson and coworkers in order to obtain very accurate energies. The CBS models use the known asymptotic convergence of pair natural orbital expressions to extrapolate from calculations using a finite basis set to the estimated complete basis set limit. CBS-4 begins with a HF/3-21G(d) geometry optimization; the zero point energy is computed at the same level. It then uses a large basis set SCF calculation as a base energy, and a MP2/6-31+G calculation with a CBS extrapolation to correct the energy through second order. A MP4(SDQ)/6-31+(d,p) calculation is used to approximate higher order contributions. In this study we applied the modified CBS-4M method (M referring to the use of minimal population localization) which is a re-parametrized version of the original CBS-4 method and also includes some additional empirical corrections. The enthalpies of the gas-phase species M were computed according to the atomization energy method (E1) (Table S6 & 7).[S13–18]

$$\Delta_f H^\circ_{(g,\ M,\ 298)} = H_{(Molecule,\ 298)} - \sum H^\circ_{(Atoms,\ 298)} + \sum \Delta_f H^\circ_{(Atoms,\ 298)} \qquad (E1)$$

**Table S6**. Literature values for atomic $\Delta H^\circ{}_f^{298}$ / kcal mol$^{-1}$

| | $-H^{298}$ [a.u.] | NIST[S19] |
|---|---|---|
| H | 0.50091 | 52.1 |
| C | 37.786156 | 171.3 |
| N | 54.522462 | 113.0 |
| O | 74.991202 | 59.6 |

The gas-phase heat of formations were converted to the solid/liquid state ones for neutrals: by subtracting the vaporization/sublimation enthalpies (calculated using the Trouton rule)[S20,21] The calculation results are summarized in Table S7.

$$\Delta U_m = \Delta H_m - \Delta n\ R\ T \qquad \text{(E2)}$$

**Table S7**. CBS-4M results, Gas phase enthalpies of formation, calculated sublimation/vaporization enthalpies and solid-state heat of formation.

| Compound | $-H^{298}$ / a.u. | $\Delta_f H°$(g) / kJ mol$^{-1}$ | $\Delta_{s/v}H°$/ kJ mol$^{-}$ | $V_m$ / nm$^3$ | $\Delta n$ |
|---|---|---|---|---|---|
| **3** | –460.528989 | 679.5 | 39.1635 | | –3.5 |
| **AET** | –499.771217 | 639.9 | 41.9535 | | *–6* |
| **APT** | –539.009606 | 775.6 | 42.1335 | | –8 |

## 4. NMR spectroscopy of **1**, **2**, and **3**

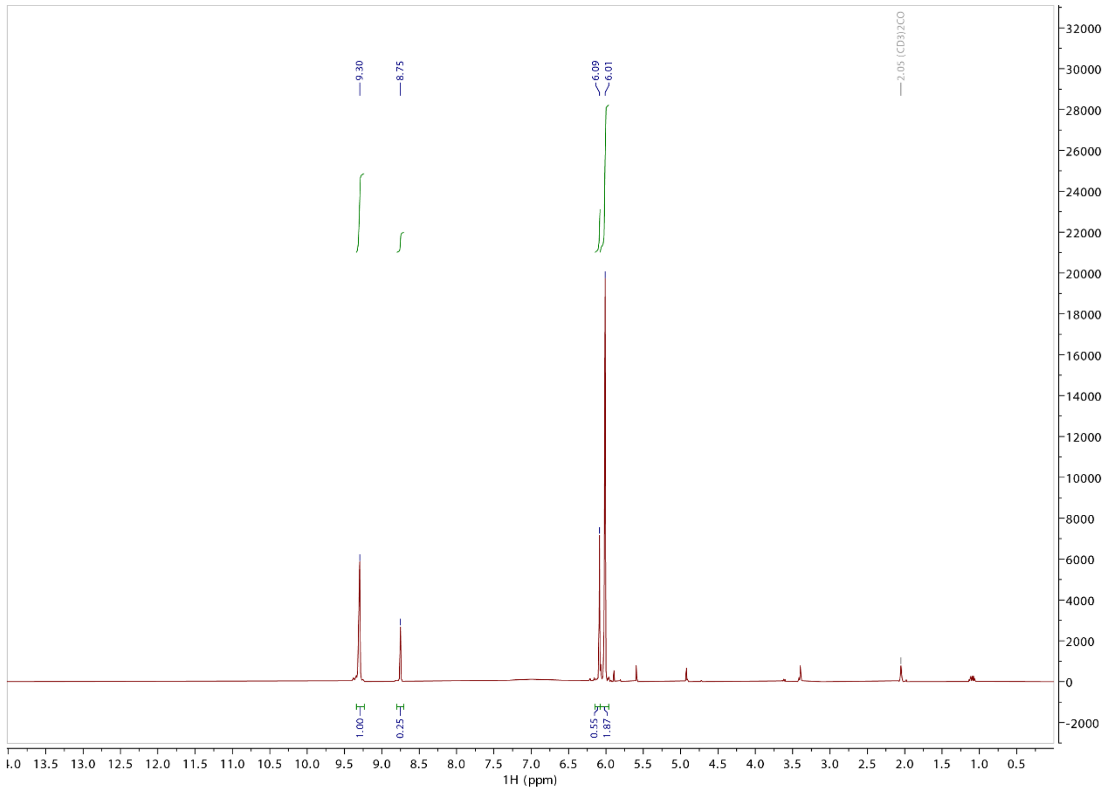


Figure S1. $^{1}$H NMR of crude isomeric mixture **1** in acetone-d$_6$.

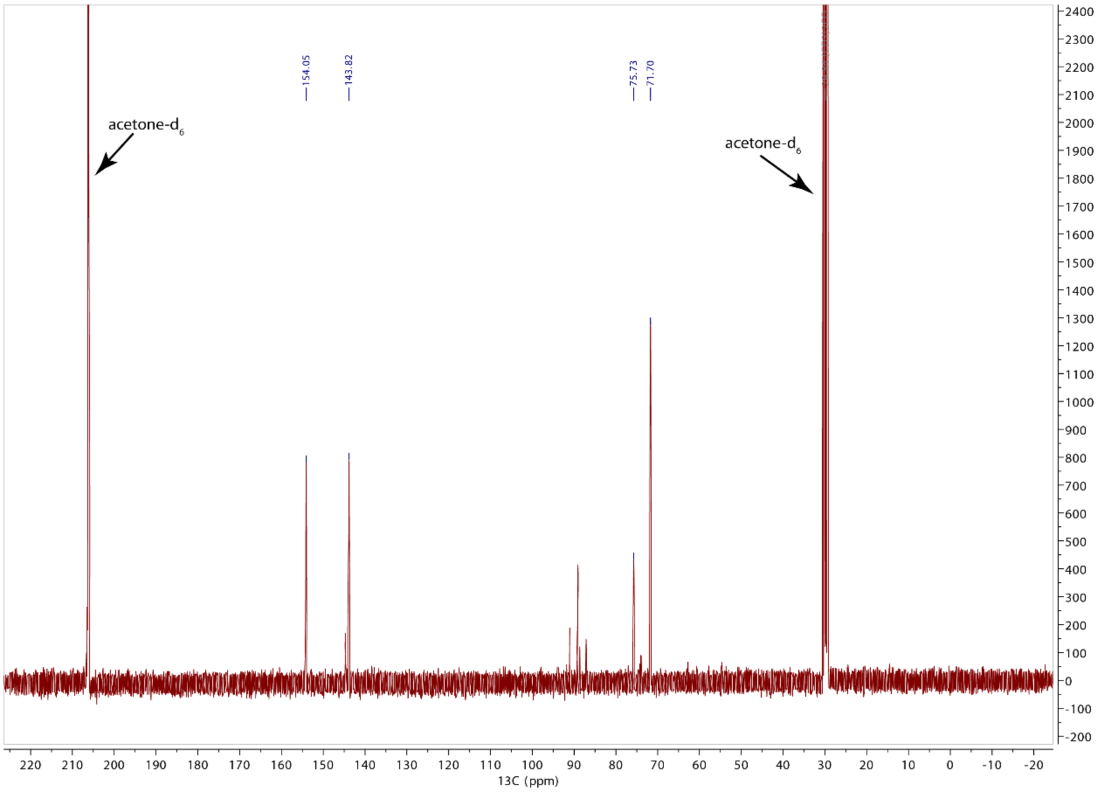


Figure S2. $^{13}$C NMR of crude isomeric mixture **1** in acetone-d$_6$.

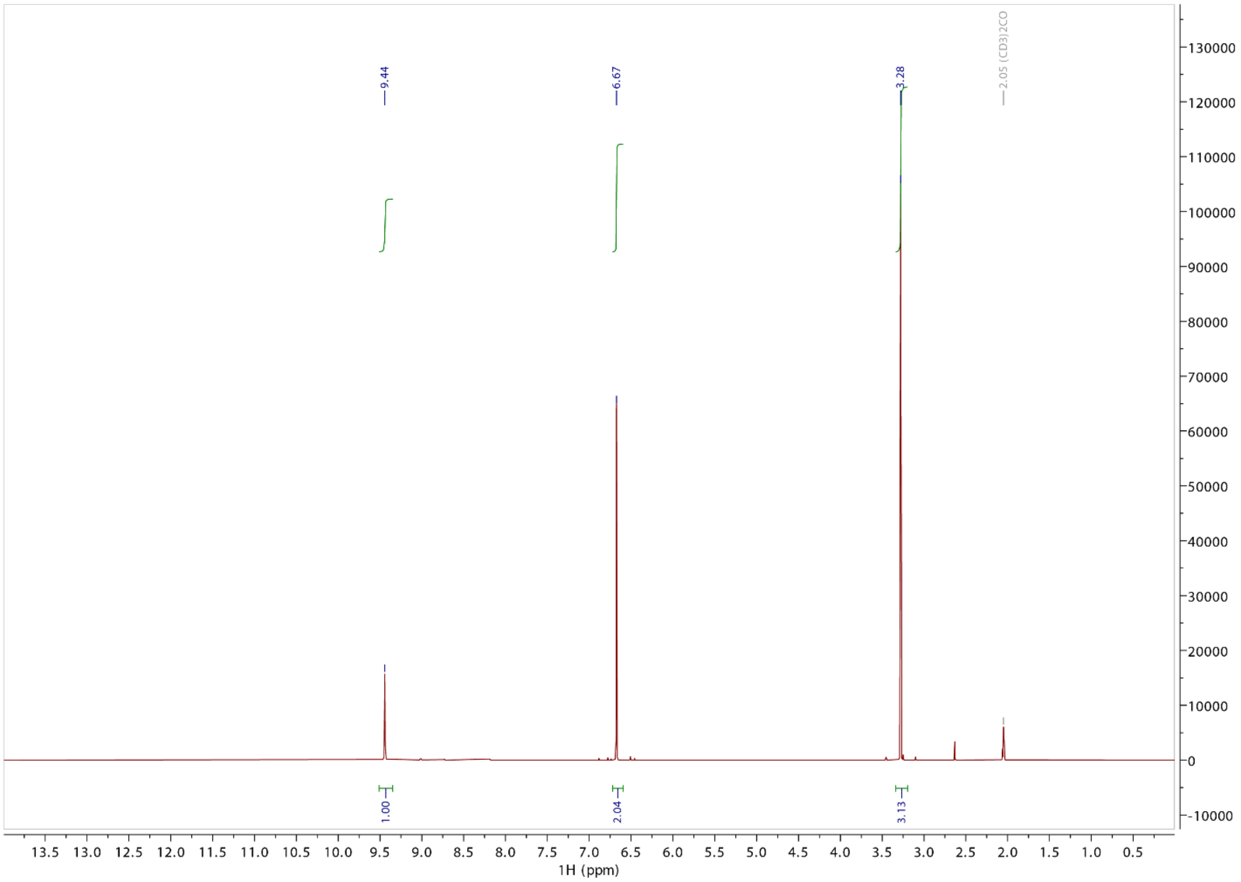


Figure S3. $^{1}$H NMR of compound **2a** in acetone-d$_6$.

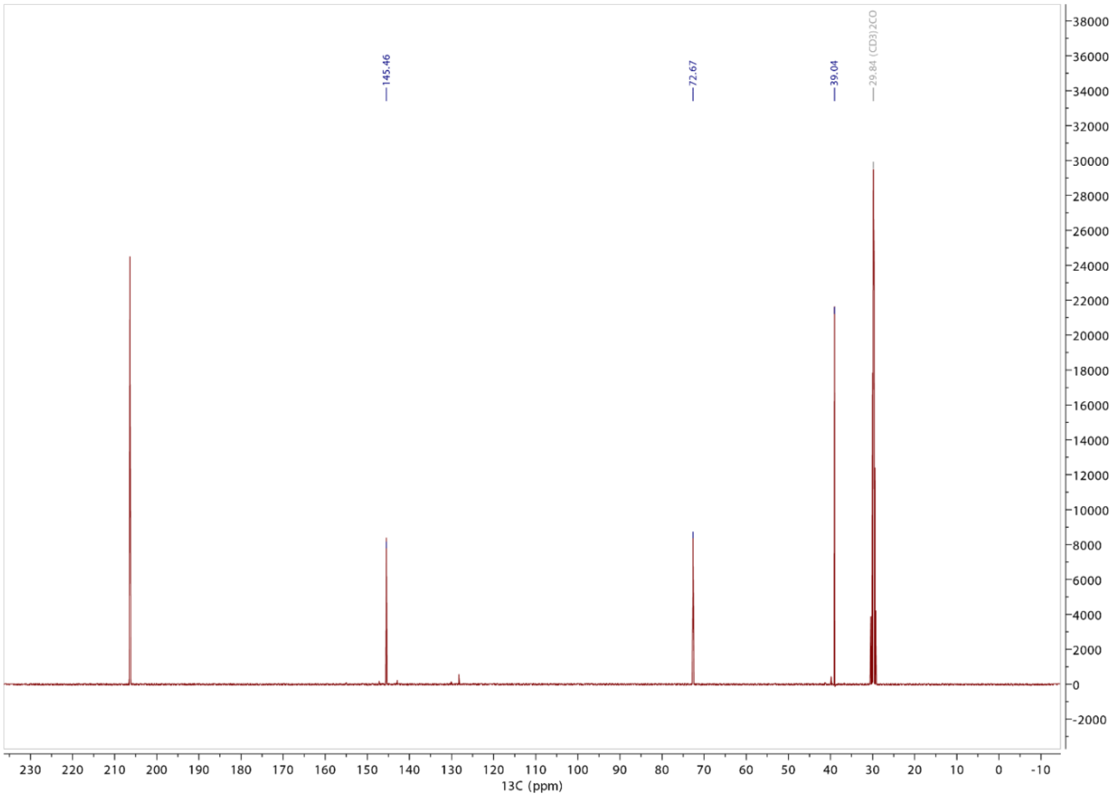


Figure S4. $^{13}$C NMR of compound **2a** in acetone-d$_6$.

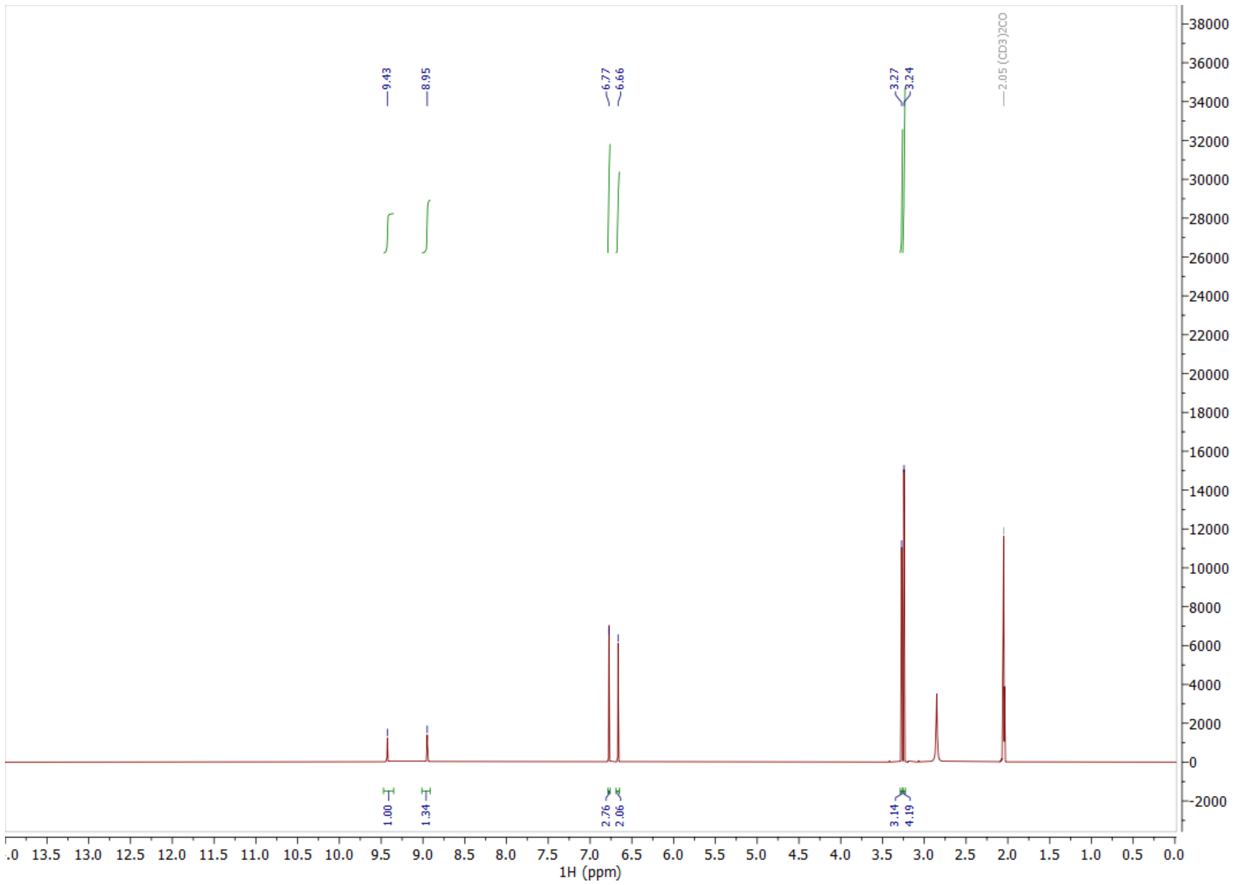


Figure S5. $^{1}$H NMR of compound **3** in DMSO-d$_6$.

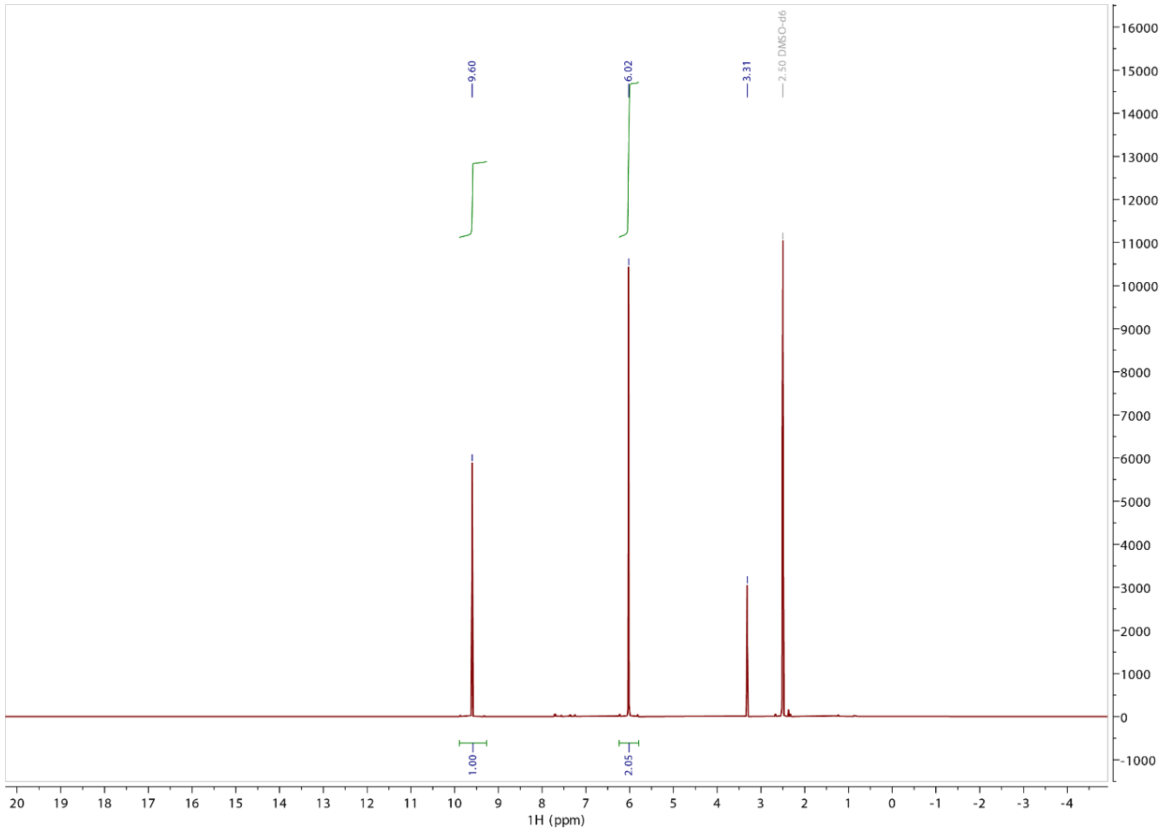

Figure S6 $^{1}$H NMR of compound **3** in DMSO-$d_6$.

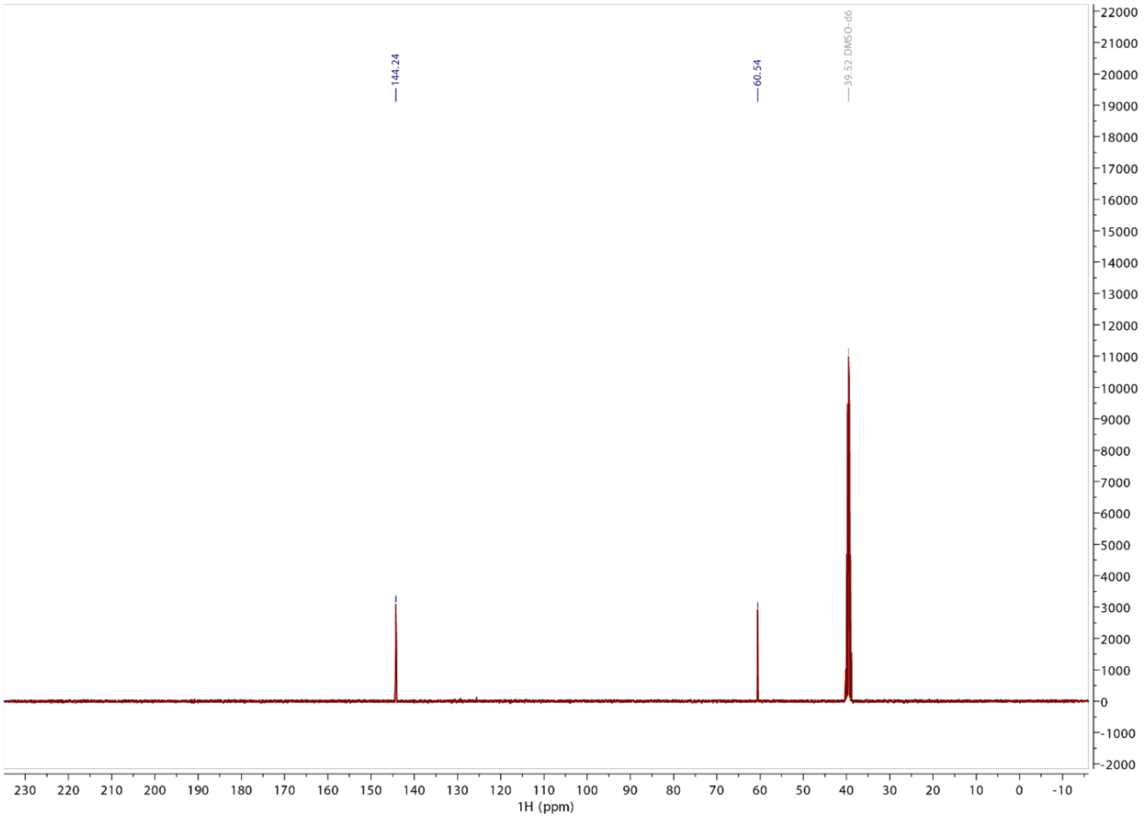


Figure S7. $^{13}$C NMR of compound **3** in DMSO-$d_6$.

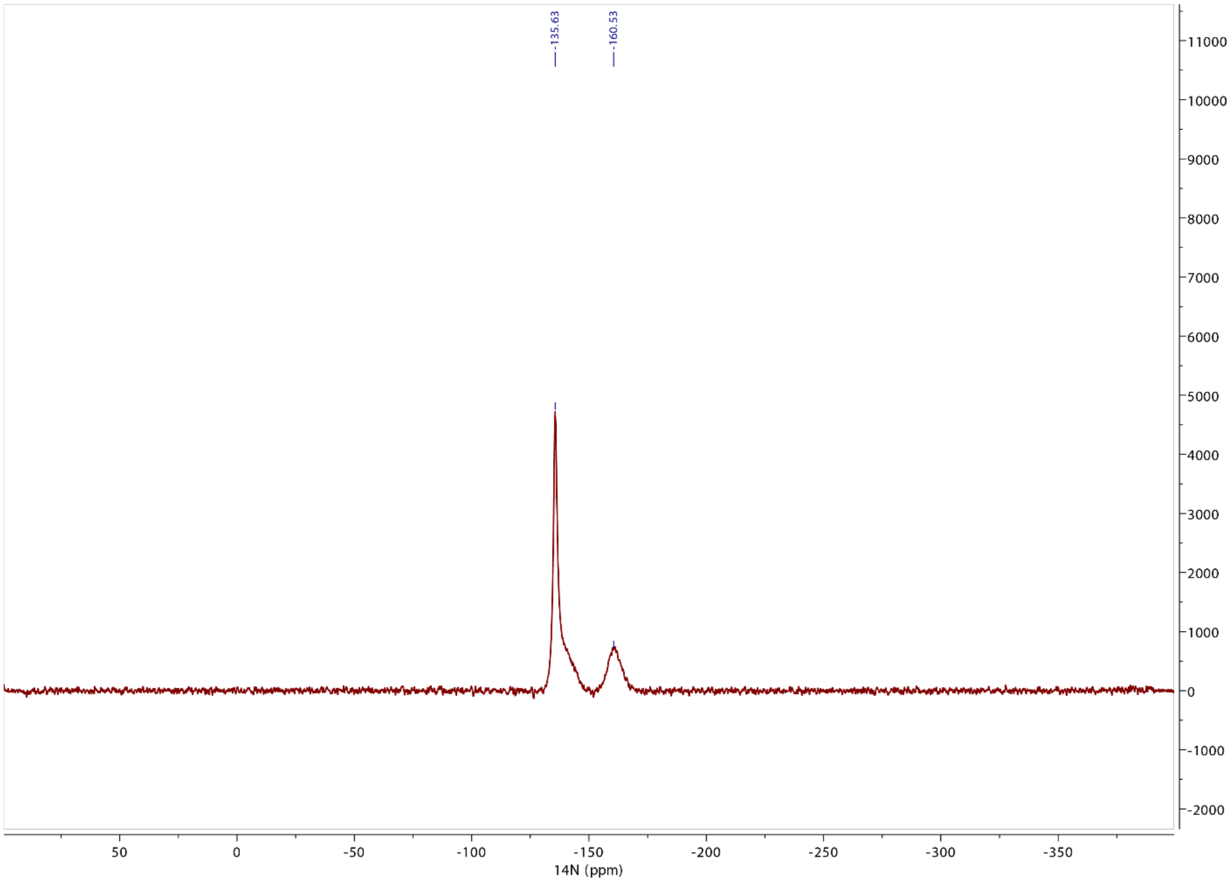


Figure S8. $^{14}N$ NMR of compound **3** in DMSO-$d_6$.

## 5. IR Spectroscopy of 1 – 15

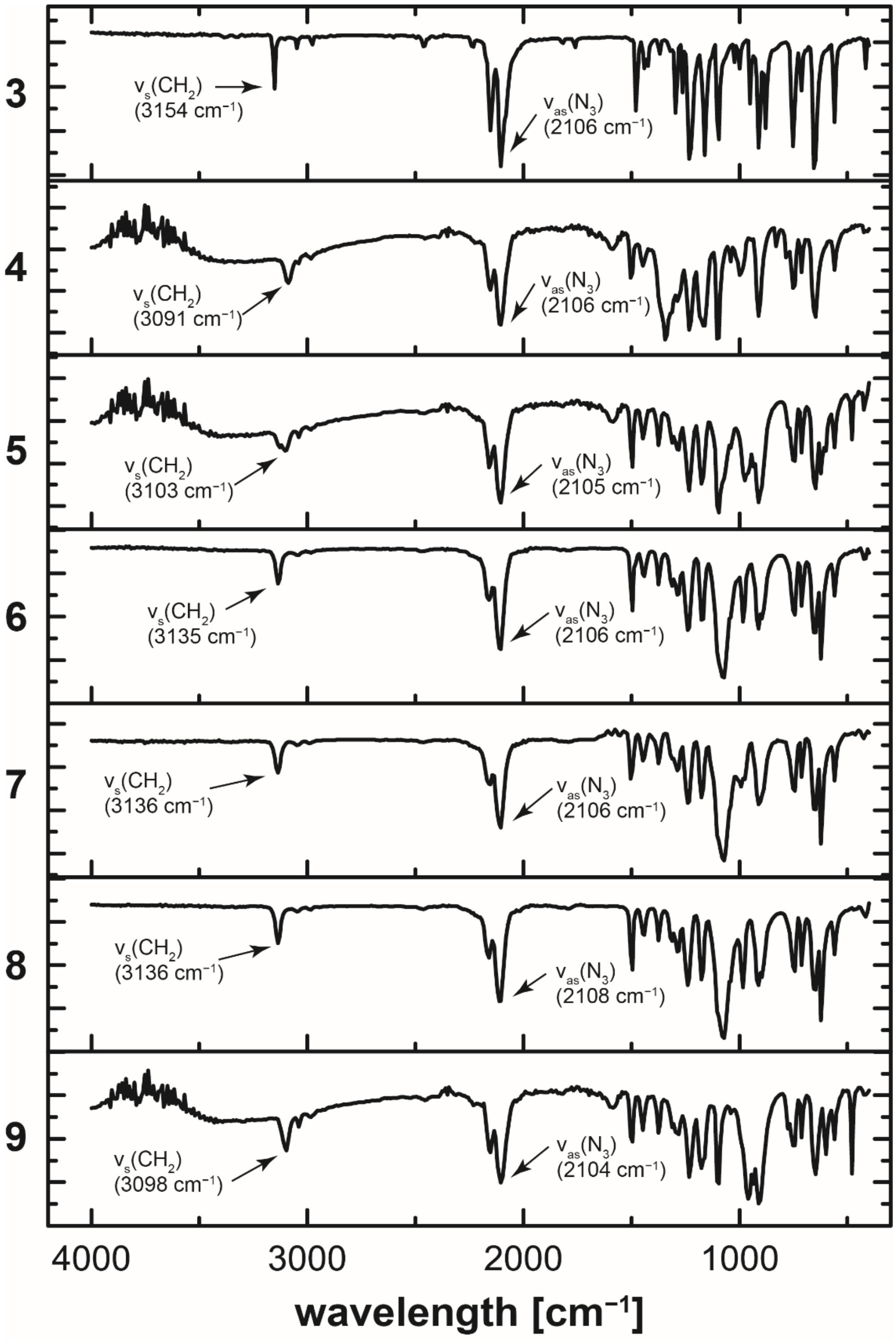


Figure S9. IR spectra of compounds **3** – **9**.

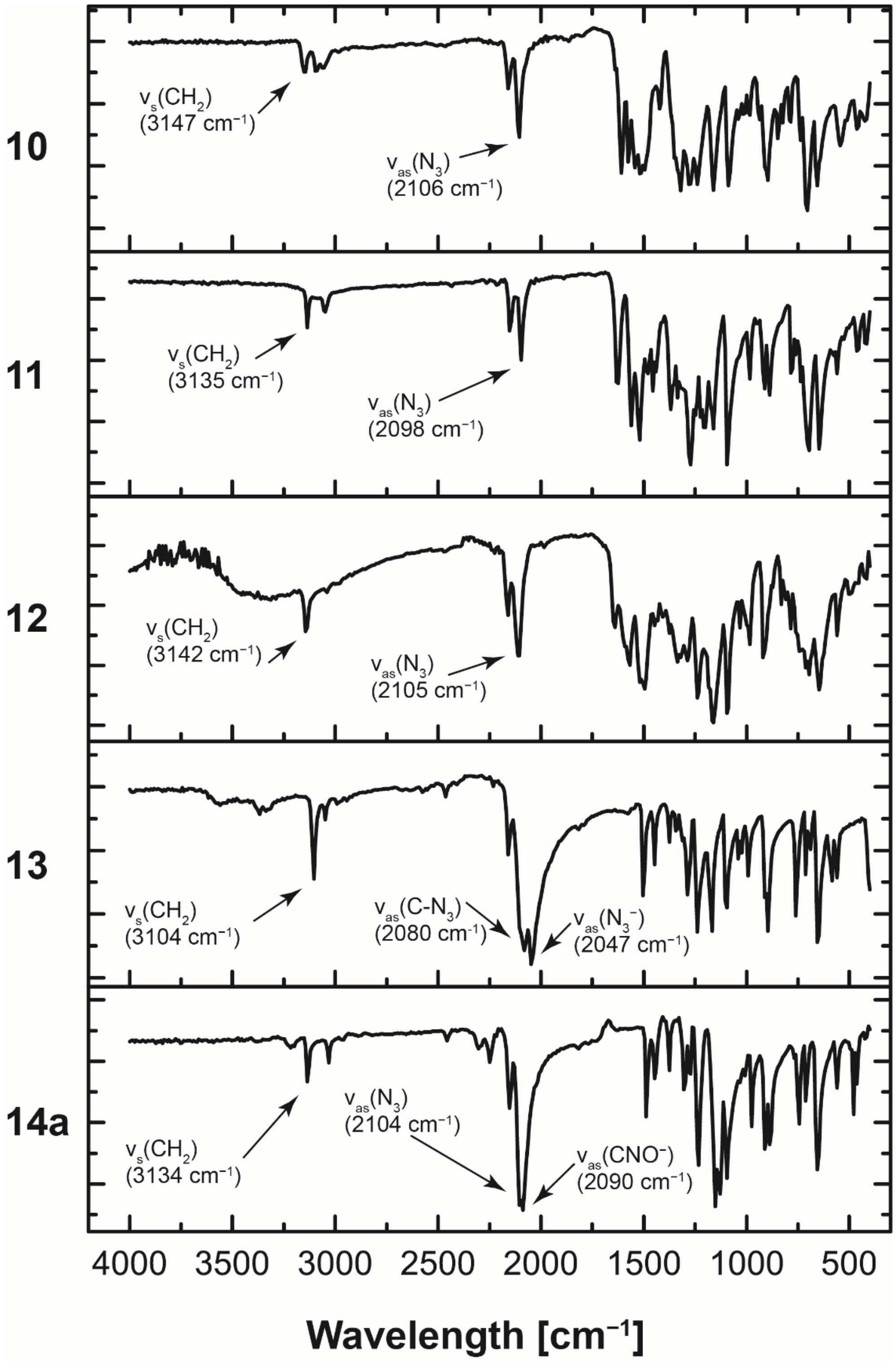


Figure S10. IR spectra of compounds **10** – **14a**.

## 6. Hot plate and hot needle test of compound 3 – 14

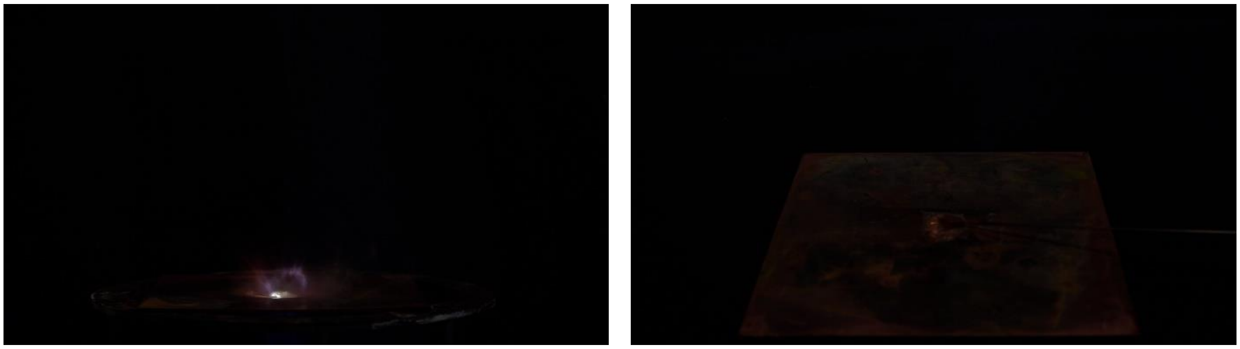

Figure S11. Hot plate (left) and hot needle (right) test of compound **3**.

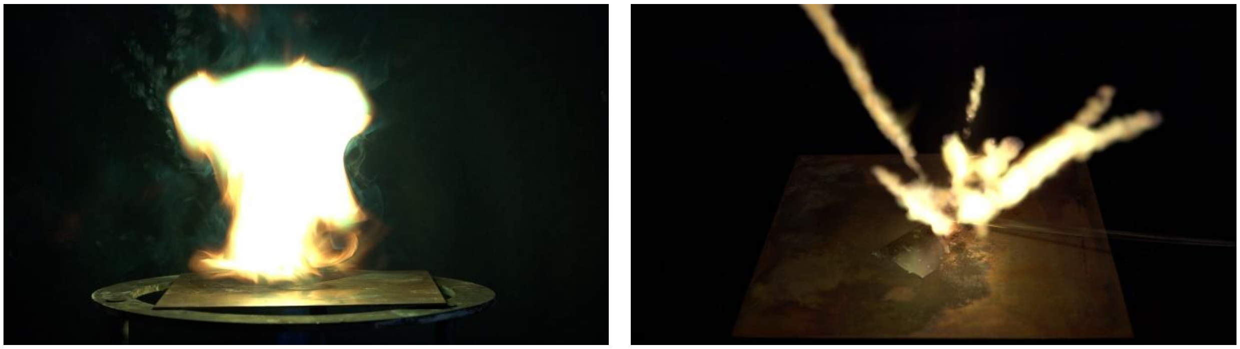

Figure S12. Hot plate (left) and hot needle (right) test of compound **4**.

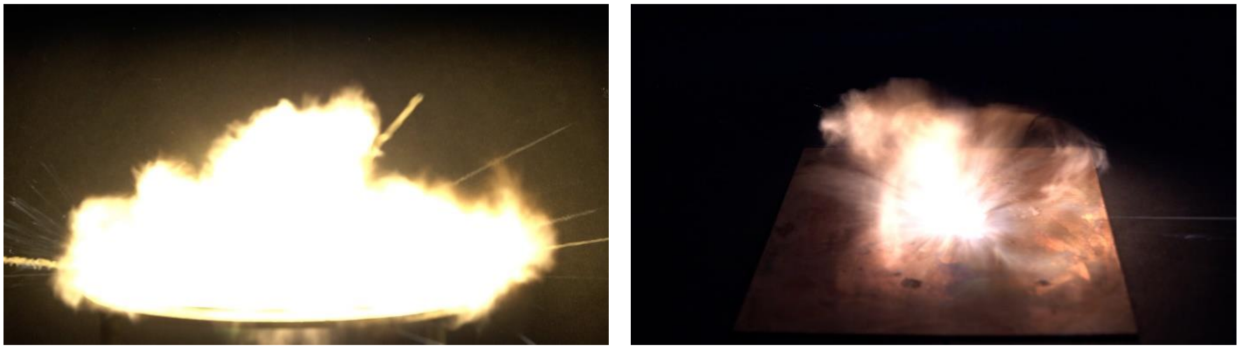

Figure S13. Hot plate (left) and hot needle (right) test of compound **6**.

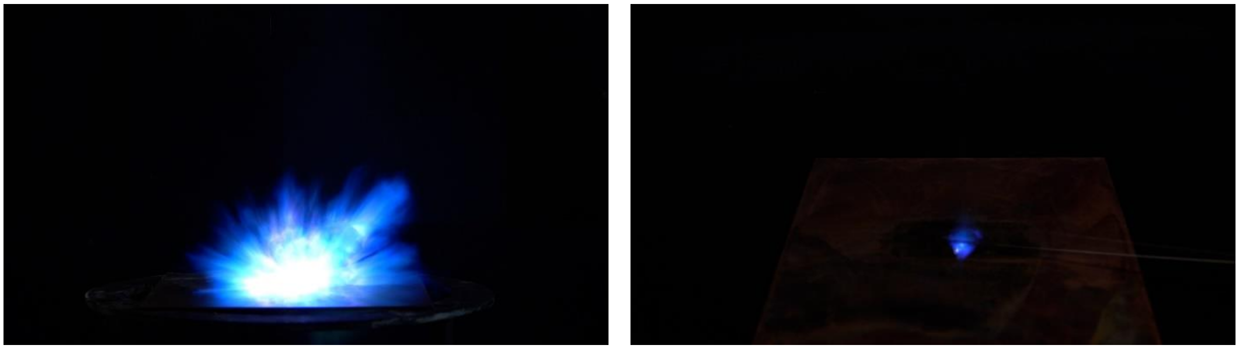

Figure S14. Hot plate (left) and hot needle (right) test of compound **7**.

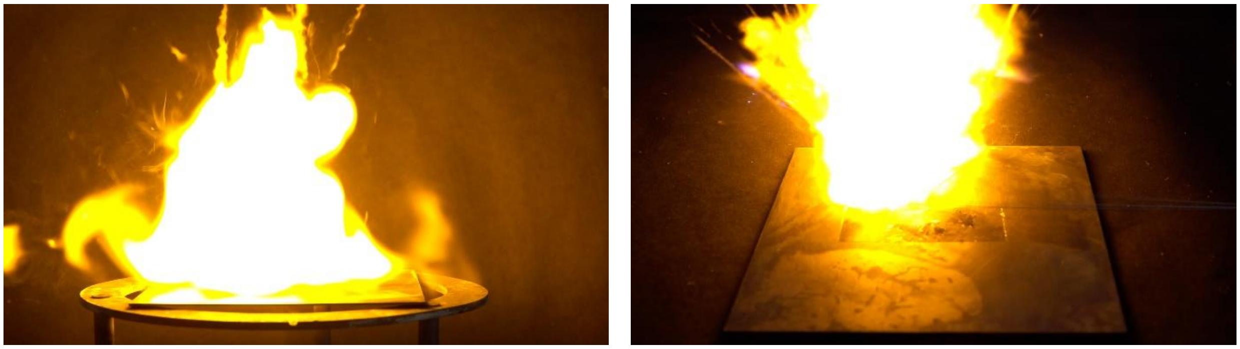
Figure S15. Hot plate (left) and hot needle (right) test of compound **8**.

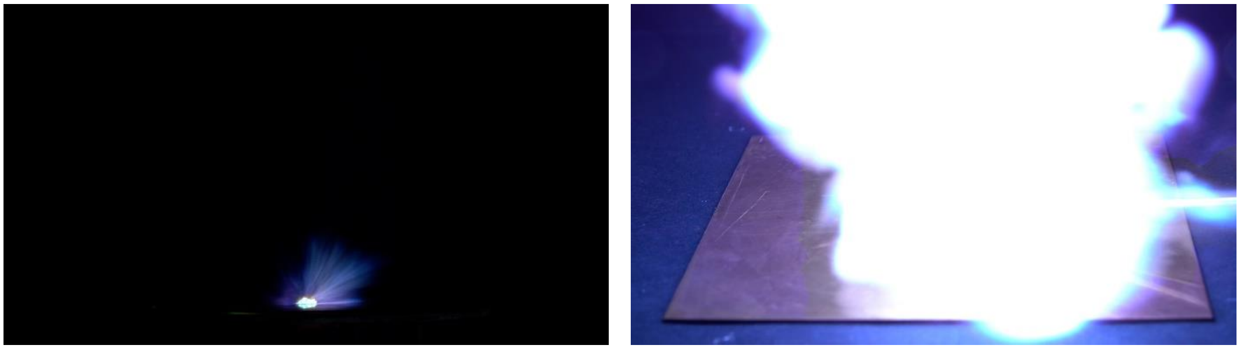
Figure S16. Hot plate (left) and hot needle (right) test of compound **9**.

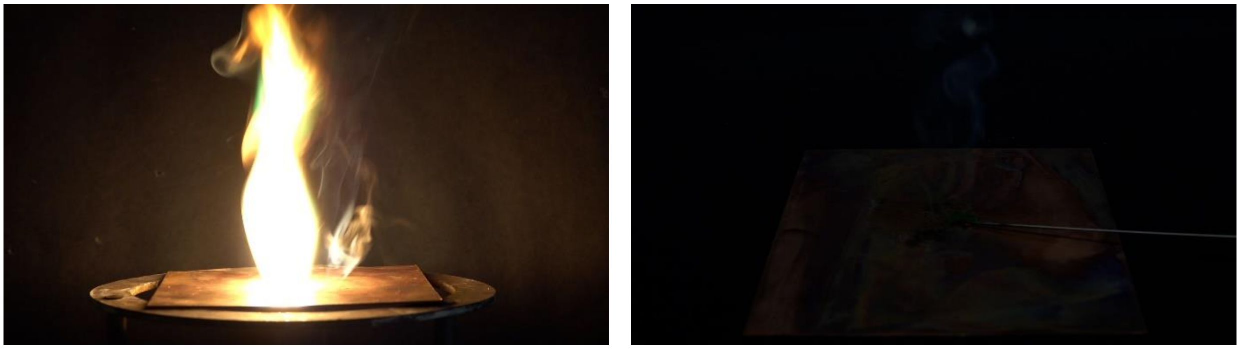
Figure S17. Hot plate (left) and hot needle (right) test of compound **10**.

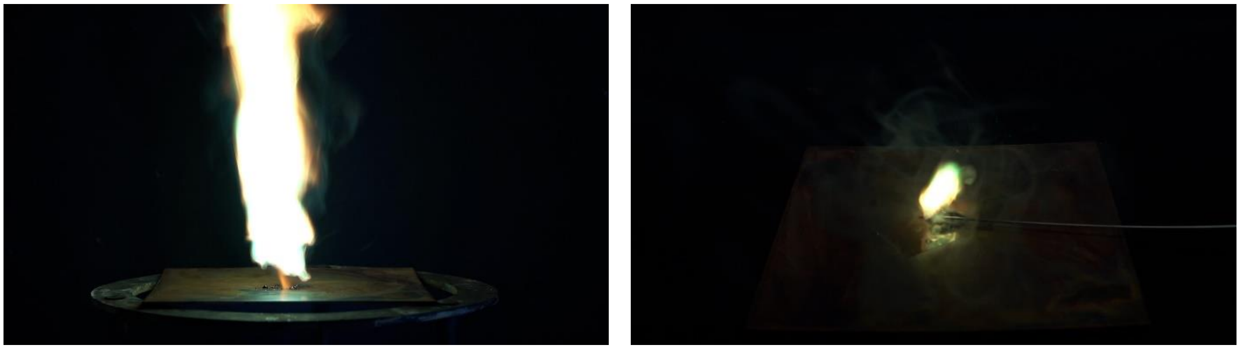
Figure S18. Hot plate (left) and hot needle (right) test of compound **11**.

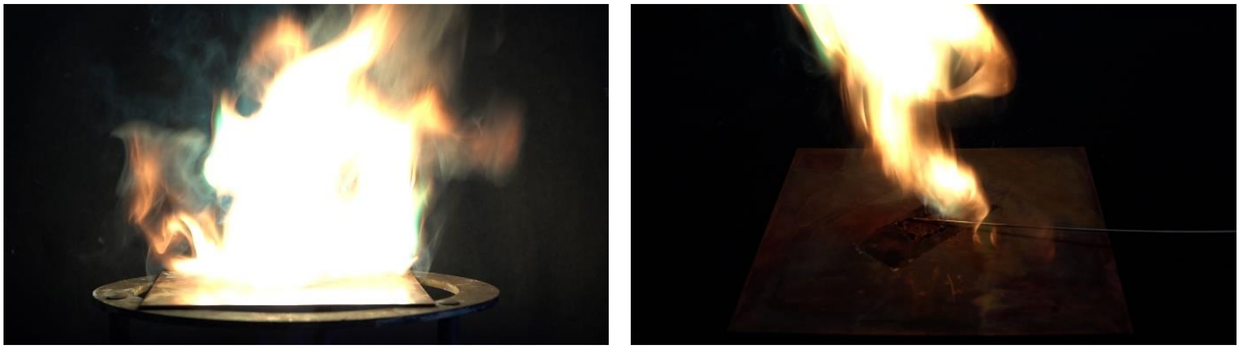

Figure S19. Hot plate (left) and hot needle (right) test of compound **12**.

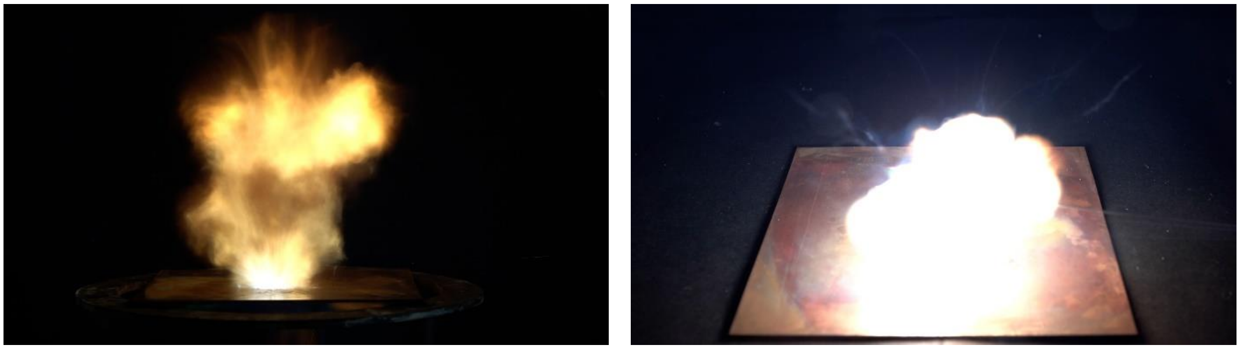

Figure S20. Hot plate (left) and hot needle (right) test of compound **13**.

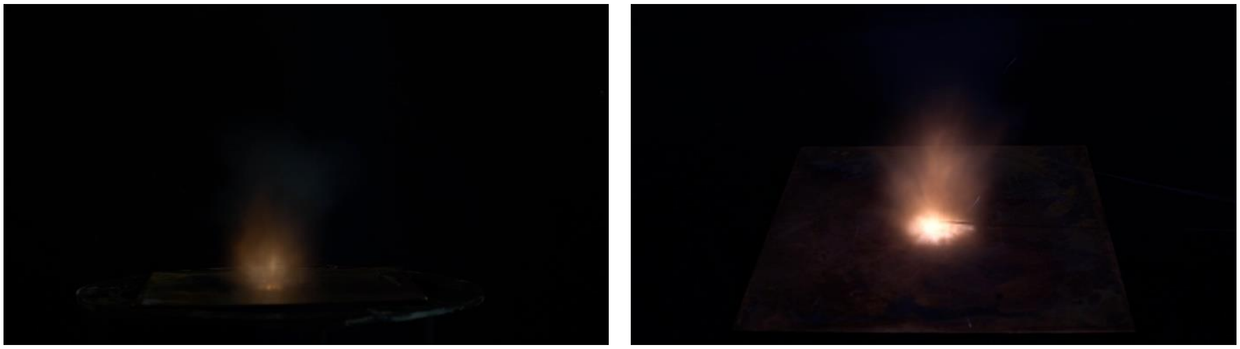

Figure S21. Hot plate (left) and hot needle (right) test of compound **14a**.

## 7. General Methods

All chemicals and solvents were employed as received (Sigma-Aldrich, Fluka, Acros, ABCR). $^{1}$H, $^{13}$C{$^{1}$H}, $^{14}$N, $^{15}$N{$^{1}$H} spectra were recorded at ambient temperature using a JEOL Bruker 27400, Eclipse 270, JEOL EX 400 or a JEOL Eclipse 400 instrument. The chemical shifts quoted in ppm in the text refer to typical standards such as tetramethylsilane ($^{1}$H, $^{13}$C) nitromethane ($^{14}$N,$^{15}$N) in DMSO-$d_6$, $D_2O$ or acetone-$d_6$ as the solvent. Endothermic and exothermic events of the described compounds, which indicate melting, loss of crystal water or decomposition, are given as the extrapolated onset temperatures. The samples were measured in a range of 25–400 °C at a heating rate of

5 °C min$^{-1}$ through differential thermal analysis (DTA) with an OZM Research DTA 552-Ex instrument. Infrared spectra were measured with pure samples on a Perkin-Elmer BXII FT-IR system with a Smith DuraSampler IR II diamond ATR. Determination of the carbon, hydrogen, and nitrogen contents was carried out by combustion analysis using an Elementar Vario El (nitrogen values determined are often lower than the calculated ones' due to their explosive behavior). Impact sensitivity tests were carried out according to STANAG 4489[S22] with a modified instruction[S23] using a BAM (Bundesanstalt für Materialforschung) drophammer.[S24] Friction sensitivity tests were carried out according to STANAG 4487[S28] with a modified instruction[S29] using the BAM friction tester.[S24,25] The classification of the tested compounds results from the "UN Recommendations on the Transport of Dangerous Goods".[S30,31] Additionally, all compounds were tested upon the sensitivity toward electrical discharge using the OZM Electric Spark XSpark10 device.[S29] Energetic properties have been calculated with the EXPLO5 6.05.04 computer code[S32] using the, to RT converted, X-ray density and calculated solid state heats of formation. These were computed by the atomization method as described in recently published papers. Electronic enthalpies were calculated with the Gaussian09 software[S13] suite using the CBS-4M method.

## 8. References


S1 CrysAlisPRO (Version 171.33.41), Oxford Diffraction Ltd., 2009.

S2 A. Altomare, G. Cascarano, C. Giacovazzo, and A. Guagliardi, *J. Appl. Crystallogr.*, 1992, **26**, 343.

S3 A. Altomare, G. Cascarano, C. Giacovazzo, A. Guagliardi, A. G. G. Moliterni, M. C. Burla, G. Polidori, M. Camalli and R. Spagna, SIR97, 2003.

S4 A. Altomare, M. C. Burla, M. Camalli, G. L. Cascarano, C. Giacovazzo, A. Guagliardi, A. G. G. Moliterni, G. Polidori and R. Spagna, *J. Appl. Crystallogr.*, 1999, **32**, 115.

S5 G. M. Sheldrick, SHELXL-97, University of Göttingen, Germany, 1997.

S6 G. M. Sheldrick, *Acta Crystallogr. Sect. A*, 2008, **64**, 112.

S7 G. M. Sheldrick, *Acta Cryst. A*, 2015, **71**, 3–8.

S8 A. L. Spek, PLATON, Utrecht University, The Netherlands, 1999.

S9 L.J. Farrugia, *J. Appl. Cryst.*, 2012, **45**, 849.

S10 O. V. Dolomanov, L. J. Bourhis, R. J. Gildea, J. A. K. Howard and H. Puschmann, *J. Appl. Cryst*., 2009, **42**, 339–341.

S11 Empirical absorption correction using spherical harmonics, implemented in SCALE3 ABSPACK scaling algorithm (CrysAlisPro Oxford Diffraction Ltd., Version 171.33.41, 2009).

S12 APEX3, Bruker AXS Inc., Madison, Wisconsin, USA.

S13 M. J. Frisch, G. W. Trucks, H. B. Schlegel, G. E. Scuseria, M. A. Robb, J. R. Cheeseman, G. Scalmani, V.Barone, B. Mennucci, G. A. Petersson, H. Nakatsuji, M. Caricato, X. Li, H.P. Hratchian, A. F. Izmaylov, J. Bloino, G. Zheng, J. L. Sonnenberg, M. Hada, M. Ehara, K. Toyota, R. Fukuda, J. Hasegawa, M. Ishida, T. Nakajima, Y. Honda, O. Kitao, H. Nakai, T. Vreven, J. A. Montgomery, Jr., J. E. Peralta, F. Ogliaro, M. Bearpark, J. J. Heyd, E. Brothers, K. N. Kudin, V. N. Staroverov, R. Kobayashi, J. Normand, K. Raghavachari, A. Rendell, J. C. Burant, S. S. Iyengar, J. Tomasi, M. Cossi, N. Rega, J. M. Millam, M.Klene, J. E. Knox, J. B. Cross, V. Bakken, C. Adamo, J. Jaramillo, R. Gomperts, R. E. Stratmann, O. Yazyev, A. J. Austin, R. Cammi, C. Pomelli, J. W. Ochterski, R. L. Martin, K. Morokuma, V. G. Zakrzewski, G. A. Voth, P. Salvador, J. J. Dannenberg, S. Dapprich, A. D. Daniels, O. Farkas, J.B. Foresman, J. V. Ortiz, J. Cioslowski and D. J. Fox, Gaussian 09 A.02, Gaussian, Inc., Wallingford, CT, USA, 2009.

S14 J. W. Ochterski, G. A. Petersson and J. A. Montgomery Jr., *J. Chem. Phys.*, 1996, **104**, 2598–2619.

S15 J. A. Montgomery Jr., M. J. Frisch, J. W. Ochterski and G. A. Petersson, *J. Chem. Phys.*, 2000, **112**, 6532–6542.

S16 L. A. Curtiss, K. Raghavachari, P. C. Redfern and J. A. Pople, *J. Chem. Phys.*, 1997, **106**, 1063–1079.

S17 E. F. C. Byrd and B. M. Rice, *J. Phys. Chem. A*, 2006, **110**, 1005–1013.

S18 B. M. Rice, S. V. Pai and J. Hare, *Comb. Flame*, 1999, **118**, 445–458.

S19 P. J. Lindstrom and W. G. Mallard, NIST Standard Reference Database Number 69, http://webbook.nist.gov/chemistry/, (accessed March 2021).

S20 M. S. Westwell, M. S. Searle, D. J. Wales and D. H. Williams, *J. Am. Chem. Soc*. 1995, **117**, 5013–5015.

S21 F. Trouton, *Philos. Mag.* 1884, **18**, 54–57.

S22 NATO standardization agreement (STANAG) on explosives, impact sensitivity tests, no. 4489, 1st ed., Sept. 17, 1999.

S23 WIWEB-Standardarbeitsanweisung 4-5.1.02, Ermittlung der Explosionsgefährlichkeit, hier der Schlagempfindlichkeit mit dem Fallhammer, Nov. 8, 2002.

S24 BAM, http://www.bam.de, (accessed March 2021).

S25 OZM, http://www.ozm.cz, (accessed March 2021).

S26 Military Standard 1751A (MIL-STD-1751A): safety and performance tests for qualification of explosives (high explosives, propellants and pyrotechnics), method 1016, Dec. 11, 2001.

S27 M. S. Gruhne, M. Lommel, M. H. H. Wurzenberger, N. Szimhradt, T. M. Klapötke and J. Stierstorfer, *Propellants Explos. Pyrotech.*, 2020, **45**, 147–153.

S28 NATO standardization agreement (STANAG) on explosive, friction sensitivity tests, no. 4487, 1st ed., Aug. 22, 2002.

S29 WIWEB-Standardarbeitsanweisung 4-5.1.03, Ermittlung der Explosionsgefährlichkeit oder der Reibeempfindlichkeit mit dem Reibeapparat, Nov. 8, 2002.

S30 UN Model Regulation: Recommendations on the Transport of Dangerous Goods – Manual of Tests and Criteria, section 13.4.2.3.3, 2015.

S31 Impact: insensitive > 40 J, less sensitive ≥ 35 J, sensitive ≥ 4 J, very sensitive ≤ 3 J; Friction: insensitive > 360 N, less sensitive = 360 N, sensitive < 360 N and > 80 N, very sensitive ≤ 80 N, extremely sensitive ≤ 10 N. According to the UN Recommendations on the Transport of Dangerous Goods, 5th ed., 2009.

S32 M. Sućeska, EXPLO5 Version 6.05 User's Guide. Zagreb, Croatia: OZM; 2018.
